# Supplementary material for: Household food insecurity and its association with academic performance among primary school adolescents in Hargeisa City, Somaliland
Source: PLoS One. 2024 Jul 12;19(7):e0303034. doi: 10.1371/journal.pone.0303034 (PMC11244796; doi:10.1371/journal.pone.0303034)
Supplement: S1 File — (DOCX) [file pone.0303034.s001.docx]

FACTOR

/VARIABLES W2WorkiRadio W3Refrigertor W4Car W8Electric W12Phone W1WorkinTV W5Bicycle W7Computer

W9Table W13Wmechine W10Bed W11GasCook W6BankAccunt

/MISSING LISTWISE

/ANALYSIS W2WorkiRadio W3Refrigertor W4Car W8Electric W12Phone W1WorkinTV W5Bicycle W7Computer

W9Table W13Wmechine W10Bed W11GasCook W6BankAccunt

/PRINT UNIVARIATE INITIAL CORRELATION KMO AIC EXTRACTION ROTATION

/FORMAT SORT BLANK(.30)

/CRITERIA MINEIGEN(1) ITERATE(25)

/EXTRACTION PC

/CRITERIA ITERATE(25)

/ROTATION VARIMAX

/METHOD=CORRELATION.

**Factor Analysis**

| **Notes** | | |
| --- | --- | --- |
| Output Created | | 05-FEB-2022 08:49:45 |
| Comments | |  |
| Input | Data | C:\Users\Admin\Documents\Sagal thesis SPSS data Analysis UPDATED\sagal MSc thesis DATA.sav |
|  | Active Dataset | DataSet1 |
|  | File Label | EPIDATA FI THESIS by SAGAL'S MSc 630 DATA |
|  | Filter | <none> |
|  | Weight | <none> |
|  | Split File | <none> |
|  | N of Rows in Working Data File | 630 |
| Missing Value Handling | Definition of Missing | MISSING=EXCLUDE: User-defined missing values are treated as missing. |
|  | Cases Used | LISTWISE: Statistics are based on cases with no missing values for any variable used. |
| Syntax | | FACTOR  /VARIABLES W2WorkiRadio W3Refrigertor W4Car W8Electric W12Phone W1WorkinTV W5Bicycle W7Computer  W9Table W13Wmechine W10Bed W11GasCook W6BankAccunt  /MISSING LISTWISE  /ANALYSIS W2WorkiRadio W3Refrigertor W4Car W8Electric W12Phone W1WorkinTV W5Bicycle W7Computer  W9Table W13Wmechine W10Bed W11GasCook W6BankAccunt  /PRINT UNIVARIATE INITIAL CORRELATION KMO AIC EXTRACTION ROTATION  /FORMAT SORT BLANK(.30)  /PLOT EIGEN  /CRITERIA MINEIGEN(1) ITERATE(25)  /EXTRACTION PC  /CRITERIA ITERATE(25)  /ROTATION VARIMAX  /METHOD=CORRELATION. |
| Resources | Processor Time | 00:00:00.39 |
|  | Elapsed Time | 00:00:00.38 |
|  | Maximum Memory Required | 21944 (21.430K) bytes |

| **Descriptive Statistics** | | | |
| --- | --- | --- | --- |
|  | Mean | Std. Deviation | Analysis N |
| Radio | .26 | .440 | 630 |
| Refrigerator | .50 | .500 | 630 |
| Car | .21 | .407 | 630 |
| Electricity | .98 | .148 | 630 |
| Phone | .99 | .079 | 630 |
| TV | .75 | .434 | 630 |
| Bicycle | .18 | .385 | 630 |
| Computer | .52 | .500 | 630 |
| Table and chair | .52 | .500 | 630 |
| Wash mechine | .76 | .426 | 630 |
| Bed | .81 | .393 | 630 |
| Gas cooker | .54 | .499 | 630 |
| Bank account | .40 | .490 | 630 |

| **Correlation Matrix** | | | | | | | | | | | | | | |
| --- | --- | --- | --- | --- | --- | --- | --- | --- | --- | --- | --- | --- | --- | --- |
|  | | Radio | Refrigerator | Car | Electricity | Phone | TV | Bicycle | Computer | Table and chair | Wash mechine | Bed | Gas cooker | Bank account |
| Correlation | Radio | 1.000 | .596 | .598 | -.057 | -.043 | .345 | .245 | .348 | .348 | .265 | .215 | .123 | .196 |
|  | Refrigerator | .596 | 1.000 | .515 | -.022 | -.080 | -.242 | .148 | .235 | .261 | .164 | .202 | .223 | .130 |
|  | Car | .598 | .515 | 1.000 | -.134 | -.057 | .028 | .366 | .338 | .307 | .233 | .190 | .178 | .109 |
|  | Electricity | -.057 | -.022 | -.134 | 1.000 | .259 | .012 | -.041 | -.058 | -.145 | .017 | .037 | .034 | .078 |
|  | Phone | -.043 | -.080 | -.057 | .259 | 1.000 | .046 | -.170 | .003 | -.077 | .049 | .063 | -.074 | -.017 |
|  | TV | .345 | -.242 | .028 | .012 | .046 | 1.000 | -.090 | -.101 | -.101 | .192 | -.001 | -.064 | .005 |
|  | Bicycle | .245 | .148 | .366 | -.041 | -.170 | -.090 | 1.000 | .253 | .236 | .108 | .165 | .136 | .108 |
|  | Computer | .348 | .235 | .338 | -.058 | .003 | -.101 | .253 | 1.000 | .402 | .105 | .117 | .236 | .129 |
|  | Table and chair | .348 | .261 | .307 | -.145 | -.077 | -.101 | .236 | .402 | 1.000 | .254 | .247 | .236 | .298 |
|  | Wash mechine | .265 | .164 | .233 | .017 | .049 | .192 | .108 | .105 | .254 | 1.000 | .868 | .112 | .240 |
|  | Bed | .215 | .202 | .190 | .037 | .063 | -.001 | .165 | .117 | .247 | .868 | 1.000 | .152 | .245 |
|  | Gas cooker | .123 | .223 | .178 | .034 | -.074 | -.064 | .136 | .236 | .236 | .112 | .152 | 1.000 | .163 |
|  | Bank account | .196 | .130 | .109 | .078 | -.017 | .005 | .108 | .129 | .298 | .240 | .245 | .163 | 1.000 |

| **KMO and Bartlett's Test** | | |
| --- | --- | --- |
| Kaiser-Meyer-Olkin Measure of Sampling Adequacy. | | .570 |
| Bartlett's Test of Sphericity | Approx. Chi-Square | 2766.782 |
|  | df | 78 |
|  | Sig. | .000 |

| **Anti-image Matrices** | | | | | | | | | | | | | | |
| --- | --- | --- | --- | --- | --- | --- | --- | --- | --- | --- | --- | --- | --- | --- |
|  | | Radio | Refrigerator | Car | Electricity | Phone | TV | Bicycle | Computer | Table and chair | Wash mechine | Bed | Gas cooker | Bank account |
| Anti-image Covariance | Radio | .266 | -.202 | -.107 | -.002 | -.001 | -.224 | -.052 | -.105 | -.086 | .030 | -.029 | .076 | -.044 |
|  | Refrigerator | -.202 | .367 | -.074 | -.031 | .037 | .231 | .092 | .074 | .045 | -.022 | .010 | -.103 | .019 |
|  | Car | -.107 | -.074 | .518 | .088 | -.040 | .035 | -.168 | -.051 | 4.841E-5 | -.050 | .041 | -.040 | .034 |
|  | Electricity | -.002 | -.031 | .088 | .878 | -.229 | -.005 | -.052 | .006 | .110 | -.003 | -.003 | -.063 | -.096 |
|  | Phone | -.001 | .037 | -.040 | -.229 | .880 | -.005 | .150 | -.059 | .014 | .014 | -.034 | .063 | .024 |
|  | TV | -.224 | .231 | .035 | -.005 | -.005 | .422 | .071 | .110 | .107 | -.104 | .090 | -.072 | .023 |
|  | Bicycle | -.052 | .092 | -.168 | -.052 | .150 | .071 | .768 | -.057 | -.036 | .036 | -.051 | -.029 | -.009 |
|  | Computer | -.105 | .074 | -.051 | .006 | -.059 | .110 | -.057 | .712 | -.150 | -.009 | .016 | -.128 | .019 |
|  | Table and chair | -.086 | .045 | 4.841E-5 | .110 | .014 | .107 | -.036 | -.150 | .662 | -.047 | .019 | -.096 | -.150 |
|  | Wash mechine | .030 | -.022 | -.050 | -.003 | .014 | -.104 | .036 | -.009 | -.047 | .197 | -.176 | .031 | -.019 |
|  | Bed | -.029 | .010 | .041 | -.003 | -.034 | .090 | -.051 | .016 | .019 | -.176 | .206 | -.041 | -.016 |
|  | Gas cooker | .076 | -.103 | -.040 | -.063 | .063 | -.072 | -.029 | -.128 | -.096 | .031 | -.041 | .854 | -.073 |
|  | Bank account | -.044 | .019 | .034 | -.096 | .024 | .023 | -.009 | .019 | -.150 | -.019 | -.016 | -.073 | .851 |
| Anti-image Correlation | Radio | .537^a^ | -.646 | -.288 | -.004 | -.003 | -.667 | -.116 | -.240 | -.206 | .133 | -.124 | .160 | -.092 |
|  | Refrigerator | -.646 | .517^a^ | -.169 | -.055 | .065 | .587 | .174 | .144 | .092 | -.081 | .036 | -.184 | .035 |
|  | Car | -.288 | -.169 | .810^a^ | .130 | -.060 | .074 | -.267 | -.084 | 8.269E-5 | -.157 | .125 | -.060 | .051 |
|  | Electricity | -.004 | -.055 | .130 | .486^a^ | -.261 | -.009 | -.063 | .007 | .145 | -.007 | -.006 | -.073 | -.112 |
|  | Phone | -.003 | .065 | -.060 | -.261 | .497^a^ | -.009 | .183 | -.074 | .018 | .034 | -.081 | .073 | .028 |
|  | TV | -.667 | .587 | .074 | -.009 | -.009 | .181^a^ | .125 | .201 | .203 | -.362 | .304 | -.119 | .038 |
|  | Bicycle | -.116 | .174 | -.267 | -.063 | .183 | .125 | .686^a^ | -.077 | -.050 | .092 | -.128 | -.036 | -.011 |
|  | Computer | -.240 | .144 | -.084 | .007 | -.074 | .201 | -.077 | .745^a^ | -.218 | -.024 | .041 | -.164 | .024 |
|  | Table and chair | -.206 | .092 | 8.269E-5 | .145 | .018 | .203 | -.050 | -.218 | .771^a^ | -.130 | .052 | -.128 | -.199 |
|  | Wash mechine | .133 | -.081 | -.157 | -.007 | .034 | -.362 | .092 | -.024 | -.130 | .530^a^ | -.872 | .077 | -.046 |
|  | Bed | -.124 | .036 | .125 | -.006 | -.081 | .304 | -.128 | .041 | .052 | -.872 | .536^a^ | -.098 | -.039 |
|  | Gas cooker | .160 | -.184 | -.060 | -.073 | .073 | -.119 | -.036 | -.164 | -.128 | .077 | -.098 | .658^a^ | -.086 |
|  | Bank account | -.092 | .035 | .051 | -.112 | .028 | .038 | -.011 | .024 | -.199 | -.046 | -.039 | -.086 | .810^a^ |
| a. Measures of Sampling Adequacy(MSA) | | | | | | | | | | | | | | |

| **Communalities** | | |
| --- | --- | --- |
|  | Initial | Extraction |
| Radio | 1.000 | .870 |
| Refrigerator | 1.000 | .756 |
| Car | 1.000 | .706 |
| Electricity | 1.000 | .622 |
| Phone | 1.000 | .592 |
| TV | 1.000 | .935 |
| Bicycle | 1.000 | .306 |
| Computer | 1.000 | .488 |
| Table and chair | 1.000 | .556 |
| Wash mechine | 1.000 | .928 |
| Bed | 1.000 | .932 |
| Gas cooker | 1.000 | .388 |
| Bank account | 1.000 | .514 |
| Extraction Method: Principal Component Analysis. | | |

| **Total Variance Explained** | | | | | | | | | |
| --- | --- | --- | --- | --- | --- | --- | --- | --- | --- |
| Component | Initial Eigenvalues | | | Extraction Sums of Squared Loadings | | | Rotation Sums of Squared Loadings | | |
|  | Total | % of Variance | Cumulative % | Total | % of Variance | Cumulative % | Total | % of Variance | Cumulative % |
| 1 | 3.373 | 25.947 | 25.947 | 3.373 | 25.947 | 25.947 | 2.302 | 17.710 | 17.710 |
| 2 | 1.702 | 13.089 | 39.035 | 1.702 | 13.089 | 39.035 | 1.908 | 14.675 | 32.384 |
| 3 | 1.313 | 10.099 | 49.135 | 1.313 | 10.099 | 49.135 | 1.791 | 13.780 | 46.165 |
| 4 | 1.202 | 9.248 | 58.382 | 1.202 | 9.248 | 58.382 | 1.332 | 10.250 | 56.414 |
| 5 | 1.002 | 7.711 | 66.093 | 1.002 | 7.711 | 66.093 | 1.258 | 9.679 | 66.093 |
| 6 | .896 | 6.890 | 72.983 |  |  |  |  |  |  |
| 7 | .885 | 6.808 | 79.792 |  |  |  |  |  |  |
| 8 | .828 | 6.373 | 86.164 |  |  |  |  |  |  |
| 9 | .623 | 4.795 | 90.960 |  |  |  |  |  |  |
| 10 | .527 | 4.056 | 95.015 |  |  |  |  |  |  |
| 11 | .392 | 3.018 | 98.034 |  |  |  |  |  |  |
| 12 | .158 | 1.217 | 99.251 |  |  |  |  |  |  |
| 13 | .097 | .749 | 100.000 |  |  |  |  |  |  |
| Extraction Method: Principal Component Analysis. | | | | | | | | | |

| **Component Matrix^a^** | | | | | |
| --- | --- | --- | --- | --- | --- |
|  | Component | | | | |
|  | 1 | 2 | 3 | 4 | 5 |
| Radio | .738 |  | .528 |  |  |
| Car | .714 |  |  |  |  |
| Refrigerator | .642 |  |  |  | -.439 |
| Table and chair | .629 |  |  |  |  |
| Computer | .545 |  |  |  |  |
| Bicycle | .463 |  |  |  |  |
| Gas cooker | .388 |  | -.356 |  |  |
| Wash mechine | .578 | .704 |  |  |  |
| Bed | .576 | .659 |  |  |  |
| TV |  | .361 | .776 |  | .446 |
| Electricity |  | .351 |  | .672 |  |
| Phone |  | .396 |  | .648 |  |
| Bank account | .400 |  |  |  | .482 |
| Extraction Method: Principal Component Analysis. | | | | | |
| a. 5 components extracted. | | | | | |

| **Rotated Component Matrix^a^** | | | | | |
| --- | --- | --- | --- | --- | --- |
|  | Component | | | | |
|  | 1 | 2 | 3 | 4 | 5 |
| Car | .807 |  |  |  |  |
| Refrigerator | .806 |  |  |  |  |
| Radio | .803 |  |  |  | .417 |
| Bed |  | .944 |  |  |  |
| Wash mechine |  | .937 |  |  |  |
| Table and chair |  |  | .654 |  |  |
| Bank account |  |  | .649 |  |  |
| Gas cooker |  |  | .601 |  |  |
| Computer | .383 |  | .577 |  |  |
| Bicycle | .312 |  | .339 |  |  |
| Electricity |  |  |  | .779 |  |
| Phone |  |  |  | .760 |  |
| TV |  |  |  |  | .960 |
| Extraction Method: Principal Component Analysis.  Rotation Method: Varimax with Kaiser Normalization.^a^ | | | | | |
| a. Rotation converged in 5 iterations. | | | | | |

| **Component Transformation Matrix** | | | | | |
| --- | --- | --- | --- | --- | --- |
| Component | 1 | 2 | 3 | 4 | 5 |
| 1 | .696 | .446 | .548 | -.120 | .045 |
| 2 | -.347 | .761 | -.106 | .442 | .306 |
| 3 | .391 | -.138 | -.471 | -.103 | .772 |
| 4 | .312 | -.338 | .077 | .883 | -.054 |
| 5 | -.380 | -.298 | .679 | -.005 | .553 |
| Extraction Method: Principal Component Analysis.  Rotation Method: Varimax with Kaiser Normalization. | | | | | |

FACTOR

/VARIABLES W2WorkiRadio W3Refrigertor W4Car W8Electric W12Phone W5Bicycle W7Computer W9Table

W13Wmechine W10Bed W11GasCook W6BankAccunt

/MISSING LISTWISE

/ANALYSIS W2WorkiRadio W3Refrigertor W4Car W8Electric W12Phone W5Bicycle W7Computer W9Table

W13Wmechine W10Bed W11GasCook W6BankAccunt

/PRINT UNIVARIATE INITIAL CORRELATION KMO AIC EXTRACTION ROTATION

/FORMAT SORT BLANK(.30)

/CRITERIA MINEIGEN(1) ITERATE(25)

/EXTRACTION PC

/CRITERIA ITERATE(25)

/ROTATION VARIMAX

/METHOD=CORRELATION.

**Factor Analysis**

| **Notes** | | |
| --- | --- | --- |
| Output Created | | 05-FEB-2022 08:50:29 |
| Comments | |  |
| Input | Data | C:\Users\Admin\Documents\Sagal thesis SPSS data Analysis UPDATED\sagal MSc thesis DATA.sav |
|  | Active Dataset | DataSet1 |
|  | File Label | EPIDATA FI THESIS by SAGAL'S MSc 630 DATA |
|  | Filter | <none> |
|  | Weight | <none> |
|  | Split File | <none> |
|  | N of Rows in Working Data File | 630 |
| Missing Value Handling | Definition of Missing | MISSING=EXCLUDE: User-defined missing values are treated as missing. |
|  | Cases Used | LISTWISE: Statistics are based on cases with no missing values for any variable used. |
| Syntax | | FACTOR  /VARIABLES W2WorkiRadio W3Refrigertor W4Car W8Electric W12Phone W5Bicycle W7Computer W9Table  W13Wmechine W10Bed W11GasCook W6BankAccunt  /MISSING LISTWISE  /ANALYSIS W2WorkiRadio W3Refrigertor W4Car W8Electric W12Phone W5Bicycle W7Computer W9Table  W13Wmechine W10Bed W11GasCook W6BankAccunt  /PRINT UNIVARIATE INITIAL CORRELATION KMO AIC EXTRACTION ROTATION  /FORMAT SORT BLANK(.30)  /PLOT EIGEN  /CRITERIA MINEIGEN(1) ITERATE(25)  /EXTRACTION PC  /CRITERIA ITERATE(25)  /ROTATION VARIMAX  /METHOD=CORRELATION. |
| Resources | Processor Time | 00:00:00.31 |
|  | Elapsed Time | 00:00:00.24 |
|  | Maximum Memory Required | 18976 (18.531K) bytes |

| **Descriptive Statistics** | | | |
| --- | --- | --- | --- |
|  | Mean | Std. Deviation | Analysis N |
| Radio | .26 | .440 | 630 |
| Refrigerator | .50 | .500 | 630 |
| Car | .21 | .407 | 630 |
| Electricity | .98 | .148 | 630 |
| Phone | .99 | .079 | 630 |
| Bicycle | .18 | .385 | 630 |
| Computer | .52 | .500 | 630 |
| Table and chair | .52 | .500 | 630 |
| Wash mechine | .76 | .426 | 630 |
| Bed | .81 | .393 | 630 |
| Gas cooker | .54 | .499 | 630 |
| Bank account | .40 | .490 | 630 |

| **Correlation Matrix** | | | | | | | | | | | | | |
| --- | --- | --- | --- | --- | --- | --- | --- | --- | --- | --- | --- | --- | --- |
|  | | Radio | Refrigerator | Car | Electricity | Phone | Bicycle | Computer | Table and chair | Wash mechine | Bed | Gas cooker | Bank account |
| Correlation | Radio | 1.000 | .596 | .598 | -.057 | -.043 | .245 | .348 | .348 | .265 | .215 | .123 | .196 |
|  | Refrigerator | .596 | 1.000 | .515 | -.022 | -.080 | .148 | .235 | .261 | .164 | .202 | .223 | .130 |
|  | Car | .598 | .515 | 1.000 | -.134 | -.057 | .366 | .338 | .307 | .233 | .190 | .178 | .109 |
|  | Electricity | -.057 | -.022 | -.134 | 1.000 | .259 | -.041 | -.058 | -.145 | .017 | .037 | .034 | .078 |
|  | Phone | -.043 | -.080 | -.057 | .259 | 1.000 | -.170 | .003 | -.077 | .049 | .063 | -.074 | -.017 |
|  | Bicycle | .245 | .148 | .366 | -.041 | -.170 | 1.000 | .253 | .236 | .108 | .165 | .136 | .108 |
|  | Computer | .348 | .235 | .338 | -.058 | .003 | .253 | 1.000 | .402 | .105 | .117 | .236 | .129 |
|  | Table and chair | .348 | .261 | .307 | -.145 | -.077 | .236 | .402 | 1.000 | .254 | .247 | .236 | .298 |
|  | Wash mechine | .265 | .164 | .233 | .017 | .049 | .108 | .105 | .254 | 1.000 | .868 | .112 | .240 |
|  | Bed | .215 | .202 | .190 | .037 | .063 | .165 | .117 | .247 | .868 | 1.000 | .152 | .245 |
|  | Gas cooker | .123 | .223 | .178 | .034 | -.074 | .136 | .236 | .236 | .112 | .152 | 1.000 | .163 |
|  | Bank account | .196 | .130 | .109 | .078 | -.017 | .108 | .129 | .298 | .240 | .245 | .163 | 1.000 |

| **KMO and Bartlett's Test** | | |
| --- | --- | --- |
| Kaiser-Meyer-Olkin Measure of Sampling Adequacy. | | .686 |
| Bartlett's Test of Sphericity | Approx. Chi-Square | 2230.154 |
|  | df | 66 |
|  | Sig. | .000 |

| **Anti-image Matrices** | | | | | | | | | | | | | |
| --- | --- | --- | --- | --- | --- | --- | --- | --- | --- | --- | --- | --- | --- |
|  | | Radio | Refrigerator | Car | Electricity | Phone | Bicycle | Computer | Table and chair | Wash mechine | Bed | Gas cooker | Bank account |
| Anti-image Covariance | Radio | .479 | -.218 | -.160 | -.009 | -.008 | -.027 | -.087 | -.055 | -.051 | .037 | .070 | -.057 |
|  | Refrigerator | -.218 | .560 | -.142 | -.043 | .061 | .083 | .021 | -.022 | .062 | -.066 | -.098 | .011 |
|  | Car | -.160 | -.142 | .521 | .089 | -.040 | -.178 | -.063 | -.009 | -.048 | .037 | -.035 | .032 |
|  | Electricity | -.009 | -.043 | .089 | .878 | -.229 | -.052 | .008 | .116 | -.005 | -.002 | -.065 | -.096 |
|  | Phone | -.008 | .061 | -.040 | -.229 | .880 | .154 | -.060 | .016 | .015 | -.037 | .063 | .025 |
|  | Bicycle | -.027 | .083 | -.178 | -.052 | .154 | .781 | -.080 | -.057 | .063 | -.074 | -.018 | -.013 |
|  | Computer | -.087 | .021 | -.063 | .008 | -.060 | -.080 | .742 | -.193 | .022 | -.009 | -.115 | .013 |
|  | Table and chair | -.055 | -.022 | -.009 | .116 | .016 | -.057 | -.193 | .690 | -.025 | -.004 | -.083 | -.163 |
|  | Wash mechine | -.051 | .062 | -.048 | -.005 | .015 | .063 | .022 | -.025 | .226 | -.194 | .016 | -.015 |
|  | Bed | .037 | -.066 | .037 | -.002 | -.037 | -.074 | -.009 | -.004 | -.194 | .227 | -.029 | -.023 |
|  | Gas cooker | .070 | -.098 | -.035 | -.065 | .063 | -.018 | -.115 | -.083 | .016 | -.029 | .866 | -.070 |
|  | Bank account | -.057 | .011 | .032 | -.096 | .025 | -.013 | .013 | -.163 | -.015 | -.023 | -.070 | .853 |
| Anti-image Correlation | Radio | .763^a^ | -.421 | -.321 | -.013 | -.012 | -.044 | -.146 | -.096 | -.156 | .112 | .109 | -.090 |
|  | Refrigerator | -.421 | .715^a^ | -.264 | -.061 | .087 | .125 | .033 | -.035 | .174 | -.185 | -.141 | .015 |
|  | Car | -.321 | -.264 | .779^a^ | .131 | -.059 | -.279 | -.102 | -.015 | -.140 | .108 | -.052 | .049 |
|  | Electricity | -.013 | -.061 | .131 | .481^a^ | -.261 | -.063 | .009 | .150 | -.011 | -.004 | -.075 | -.111 |
|  | Phone | -.012 | .087 | -.059 | -.261 | .485^a^ | .185 | -.074 | .020 | .034 | -.082 | .073 | .028 |
|  | Bicycle | -.044 | .125 | -.279 | -.063 | .185 | .680^a^ | -.105 | -.078 | .149 | -.176 | -.021 | -.016 |
|  | Computer | -.146 | .033 | -.102 | .009 | -.074 | -.105 | .808^a^ | -.270 | .053 | -.022 | -.144 | .017 |
|  | Table and chair | -.096 | -.035 | -.015 | .150 | .020 | -.078 | -.270 | .822^a^ | -.062 | -.011 | -.107 | -.212 |
|  | Wash mechine | -.156 | .174 | -.140 | -.011 | .034 | .149 | .053 | -.062 | .558^a^ | -.858 | .036 | -.035 |
|  | Bed | .112 | -.185 | .108 | -.004 | -.082 | -.176 | -.022 | -.011 | -.858 | .559^a^ | -.065 | -.053 |
|  | Gas cooker | .109 | -.141 | -.052 | -.075 | .073 | -.021 | -.144 | -.107 | .036 | -.065 | .766^a^ | -.082 |
|  | Bank account | -.090 | .015 | .049 | -.111 | .028 | -.016 | .017 | -.212 | -.035 | -.053 | -.082 | .807^a^ |
| a. Measures of Sampling Adequacy(MSA) | | | | | | | | | | | | | |

| **Communalities** | | |
| --- | --- | --- |
|  | Initial | Extraction |
| Radio | 1.000 | .740 |
| Refrigerator | 1.000 | .637 |
| Car | 1.000 | .706 |
| Electricity | 1.000 | .625 |
| Phone | 1.000 | .598 |
| Bicycle | 1.000 | .305 |
| Computer | 1.000 | .481 |
| Table and chair | 1.000 | .533 |
| Wash mechine | 1.000 | .918 |
| Bed | 1.000 | .906 |
| Gas cooker | 1.000 | .454 |
| Bank account | 1.000 | .436 |
| Extraction Method: Principal Component Analysis. | | |

| **Total Variance Explained** | | | | | | | | | |
| --- | --- | --- | --- | --- | --- | --- | --- | --- | --- |
| Component | Initial Eigenvalues | | | Extraction Sums of Squared Loadings | | | Rotation Sums of Squared Loadings | | |
|  | Total | % of Variance | Cumulative % | Total | % of Variance | Cumulative % | Total | % of Variance | Cumulative % |
| 1 | 3.373 | 28.107 | 28.107 | 3.373 | 28.107 | 28.107 | 2.342 | 19.518 | 19.518 |
| 2 | 1.660 | 13.830 | 41.937 | 1.660 | 13.830 | 41.937 | 1.923 | 16.026 | 35.545 |
| 3 | 1.203 | 10.025 | 51.963 | 1.203 | 10.025 | 51.963 | 1.738 | 14.485 | 50.029 |
| 4 | 1.104 | 9.197 | 61.159 | 1.104 | 9.197 | 61.159 | 1.336 | 11.130 | 61.159 |
| 5 | .897 | 7.475 | 68.634 |  |  |  |  |  |  |
| 6 | .887 | 7.395 | 76.029 |  |  |  |  |  |  |
| 7 | .843 | 7.022 | 83.051 |  |  |  |  |  |  |
| 8 | .624 | 5.197 | 88.249 |  |  |  |  |  |  |
| 9 | .532 | 4.430 | 92.679 |  |  |  |  |  |  |
| 10 | .410 | 3.420 | 96.099 |  |  |  |  |  |  |
| 11 | .350 | 2.916 | 99.014 |  |  |  |  |  |  |
| 12 | .118 | .986 | 100.000 |  |  |  |  |  |  |
| Extraction Method: Principal Component Analysis. | | | | | | | | | |

| **Component Matrix^a^** | | | | |
| --- | --- | --- | --- | --- |
|  | Component | | | |
|  | 1 | 2 | 3 | 4 |
| Radio | .736 |  |  |  |
| Car | .714 |  |  |  |
| Refrigerator | .644 |  |  |  |
| Table and chair | .630 |  |  | .337 |
| Computer | .546 |  |  | .313 |
| Bicycle | .463 |  |  |  |
| Bed | .576 | .702 |  |  |
| Wash mechine | .576 | .694 |  |  |
| Phone |  | .401 | .651 |  |
| Electricity |  | .382 | .650 |  |
| Gas cooker | .389 |  |  | .546 |
| Bank account | .400 |  |  | .456 |
| Extraction Method: Principal Component Analysis. | | | | |
| a. 4 components extracted. | | | | |

| **Rotated Component Matrix^a^** | | | | |
| --- | --- | --- | --- | --- |
|  | Component | | | |
|  | 1 | 2 | 3 | 4 |
| Radio | .835 |  |  |  |
| Car | .811 |  |  |  |
| Refrigerator | .788 |  |  |  |
| Wash mechine |  | .942 |  |  |
| Bed |  | .933 |  |  |
| Gas cooker |  |  | .670 |  |
| Table and chair |  |  | .622 |  |
| Bank account |  |  | .585 |  |
| Computer | .392 |  | .567 |  |
| Bicycle | .300 |  | .361 |  |
| Electricity |  |  |  | .778 |
| Phone |  |  |  | .761 |
| Extraction Method: Principal Component Analysis.  Rotation Method: Varimax with Kaiser Normalization.^a^ | | | | |
| a. Rotation converged in 5 iterations. | | | | |

| **Component Transformation Matrix** | | | | |
| --- | --- | --- | --- | --- |
| Component | 1 | 2 | 3 | 4 |
| 1 | .710 | .448 | .528 | -.125 |
| 2 | -.383 | .796 | -.050 | .467 |
| 3 | .362 | -.336 | .004 | .869 |
| 4 | -.467 | -.231 | .848 | .102 |
| Extraction Method: Principal Component Analysis.  Rotation Method: Varimax with Kaiser Normalization. | | | | |

FACTOR

/VARIABLES W2WorkiRadio W3Refrigertor W4Car W12Phone W5Bicycle W7Computer W9Table W13Wmechine

W10Bed W11GasCook W6BankAccunt

/MISSING LISTWISE

/ANALYSIS W2WorkiRadio W3Refrigertor W4Car W12Phone W5Bicycle W7Computer W9Table W13Wmechine

W10Bed W11GasCook W6BankAccunt

/PRINT UNIVARIATE INITIAL CORRELATION KMO AIC EXTRACTION ROTATION

/FORMAT SORT BLANK(.30)

/CRITERIA MINEIGEN(1) ITERATE(25)

/EXTRACTION PC

/CRITERIA ITERATE(25)

/ROTATION VARIMAX

/METHOD=CORRELATION.

**Factor Analysis**

| **Notes** | | |
| --- | --- | --- |
| Output Created | | 05-FEB-2022 08:51:14 |
| Comments | |  |
| Input | Data | C:\Users\Admin\Documents\Sagal thesis SPSS data Analysis UPDATED\sagal MSc thesis DATA.sav |
|  | Active Dataset | DataSet1 |
|  | File Label | EPIDATA FI THESIS by SAGAL'S MSc 630 DATA |
|  | Filter | <none> |
|  | Weight | <none> |
|  | Split File | <none> |
|  | N of Rows in Working Data File | 630 |
| Missing Value Handling | Definition of Missing | MISSING=EXCLUDE: User-defined missing values are treated as missing. |
|  | Cases Used | LISTWISE: Statistics are based on cases with no missing values for any variable used. |
| Syntax | | FACTOR  /VARIABLES W2WorkiRadio W3Refrigertor W4Car W12Phone W5Bicycle W7Computer W9Table W13Wmechine  W10Bed W11GasCook W6BankAccunt  /MISSING LISTWISE  /ANALYSIS W2WorkiRadio W3Refrigertor W4Car W12Phone W5Bicycle W7Computer W9Table W13Wmechine  W10Bed W11GasCook W6BankAccunt  /PRINT UNIVARIATE INITIAL CORRELATION KMO AIC EXTRACTION ROTATION  /FORMAT SORT BLANK(.30)  /PLOT EIGEN  /CRITERIA MINEIGEN(1) ITERATE(25)  /EXTRACTION PC  /CRITERIA ITERATE(25)  /ROTATION VARIMAX  /METHOD=CORRELATION. |
| Resources | Processor Time | 00:00:00.33 |
|  | Elapsed Time | 00:00:00.24 |
|  | Maximum Memory Required | 16224 (15.844K) bytes |

| **Descriptive Statistics** | | | |
| --- | --- | --- | --- |
|  | Mean | Std. Deviation | Analysis N |
| Radio | .26 | .440 | 630 |
| Refrigerator | .50 | .500 | 630 |
| Car | .21 | .407 | 630 |
| Phone | .99 | .079 | 630 |
| Bicycle | .18 | .385 | 630 |
| Computer | .52 | .500 | 630 |
| Table and chair | .52 | .500 | 630 |
| Wash mechine | .76 | .426 | 630 |
| Bed | .81 | .393 | 630 |
| Gas cooker | .54 | .499 | 630 |
| Bank account | .40 | .490 | 630 |

| **Correlation Matrix** | | | | | | | | | | | | |
| --- | --- | --- | --- | --- | --- | --- | --- | --- | --- | --- | --- | --- |
|  | | Radio | Refrigerator | Car | Phone | Bicycle | Computer | Table and chair | Wash mechine | Bed | Gas cooker | Bank account |
| Correlation | Radio | 1.000 | .596 | .598 | -.043 | .245 | .348 | .348 | .265 | .215 | .123 | .196 |
|  | Refrigerator | .596 | 1.000 | .515 | -.080 | .148 | .235 | .261 | .164 | .202 | .223 | .130 |
|  | Car | .598 | .515 | 1.000 | -.057 | .366 | .338 | .307 | .233 | .190 | .178 | .109 |
|  | Phone | -.043 | -.080 | -.057 | 1.000 | -.170 | .003 | -.077 | .049 | .063 | -.074 | -.017 |
|  | Bicycle | .245 | .148 | .366 | -.170 | 1.000 | .253 | .236 | .108 | .165 | .136 | .108 |
|  | Computer | .348 | .235 | .338 | .003 | .253 | 1.000 | .402 | .105 | .117 | .236 | .129 |
|  | Table and chair | .348 | .261 | .307 | -.077 | .236 | .402 | 1.000 | .254 | .247 | .236 | .298 |
|  | Wash mechine | .265 | .164 | .233 | .049 | .108 | .105 | .254 | 1.000 | .868 | .112 | .240 |
|  | Bed | .215 | .202 | .190 | .063 | .165 | .117 | .247 | .868 | 1.000 | .152 | .245 |
|  | Gas cooker | .123 | .223 | .178 | -.074 | .136 | .236 | .236 | .112 | .152 | 1.000 | .163 |
|  | Bank account | .196 | .130 | .109 | -.017 | .108 | .129 | .298 | .240 | .245 | .163 | 1.000 |

| **KMO and Bartlett's Test** | | |
| --- | --- | --- |
| Kaiser-Meyer-Olkin Measure of Sampling Adequacy. | | .697 |
| Bartlett's Test of Sphericity | Approx. Chi-Square | 2150.115 |
|  | df | 55 |
|  | Sig. | .000 |

| **Anti-image Matrices** | | | | | | | | | | | | |
| --- | --- | --- | --- | --- | --- | --- | --- | --- | --- | --- | --- | --- |
|  | | Radio | Refrigerator | Car | Phone | Bicycle | Computer | Table and chair | Wash mechine | Bed | Gas cooker | Bank account |
| Anti-image Covariance | Radio | .479 | -.220 | -.162 | -.011 | -.027 | -.087 | -.055 | -.051 | .037 | .070 | -.059 |
|  | Refrigerator | -.220 | .562 | -.141 | .054 | .081 | .022 | -.017 | .062 | -.066 | -.103 | .006 |
|  | Car | -.162 | -.141 | .530 | -.019 | -.176 | -.065 | -.022 | -.048 | .038 | -.029 | .043 |
|  | Phone | -.011 | .054 | -.019 | .945 | .151 | -.062 | .051 | .015 | -.040 | .050 | -.001 |
|  | Bicycle | -.027 | .081 | -.176 | .151 | .784 | -.080 | -.052 | .063 | -.075 | -.022 | -.019 |
|  | Computer | -.087 | .022 | -.065 | -.062 | -.080 | .742 | -.198 | .022 | -.009 | -.115 | .014 |
|  | Table and chair | -.055 | -.017 | -.022 | .051 | -.052 | -.198 | .706 | -.024 | -.004 | -.076 | -.155 |
|  | Wash mechine | -.051 | .062 | -.048 | .015 | .063 | .022 | -.024 | .226 | -.195 | .016 | -.016 |
|  | Bed | .037 | -.066 | .038 | -.040 | -.075 | -.009 | -.004 | -.195 | .227 | -.029 | -.024 |
|  | Gas cooker | .070 | -.103 | -.029 | .050 | -.022 | -.115 | -.076 | .016 | -.029 | .871 | -.079 |
|  | Bank account | -.059 | .006 | .043 | -.001 | -.019 | .014 | -.155 | -.016 | -.024 | -.079 | .863 |
| Anti-image Correlation | Radio | .761^a^ | -.423 | -.322 | -.016 | -.045 | -.146 | -.095 | -.156 | .112 | .109 | -.092 |
|  | Refrigerator | -.423 | .719^a^ | -.258 | .074 | .122 | .034 | -.026 | .173 | -.186 | -.147 | .009 |
|  | Car | -.322 | -.258 | .789^a^ | -.026 | -.274 | -.104 | -.036 | -.140 | .110 | -.043 | .064 |
|  | Phone | -.016 | .074 | -.026 | .502^a^ | .175 | -.074 | .062 | .032 | -.086 | .055 | -.001 |
|  | Bicycle | -.045 | .122 | -.274 | .175 | .692^a^ | -.104 | -.070 | .149 | -.177 | -.026 | -.024 |
|  | Computer | -.146 | .034 | -.104 | -.074 | -.104 | .805^a^ | -.274 | .053 | -.022 | -.144 | .018 |
|  | Table and chair | -.095 | -.026 | -.036 | .062 | -.070 | -.274 | .840^a^ | -.061 | -.010 | -.097 | -.199 |
|  | Wash mechine | -.156 | .173 | -.140 | .032 | .149 | .053 | -.061 | .558^a^ | -.858 | .035 | -.036 |
|  | Bed | .112 | -.186 | .110 | -.086 | -.177 | -.022 | -.010 | -.858 | .558^a^ | -.065 | -.054 |
|  | Gas cooker | .109 | -.147 | -.043 | .055 | -.026 | -.144 | -.097 | .035 | -.065 | .780^a^ | -.091 |
|  | Bank account | -.092 | .009 | .064 | -.001 | -.024 | .018 | -.199 | -.036 | -.054 | -.091 | .834^a^ |
| a. Measures of Sampling Adequacy(MSA) | | | | | | | | | | | | |

| **Communalities** | | |
| --- | --- | --- |
|  | Initial | Extraction |
| Radio | 1.000 | .740 |
| Refrigerator | 1.000 | .634 |
| Car | 1.000 | .706 |
| Phone | 1.000 | .798 |
| Bicycle | 1.000 | .465 |
| Computer | 1.000 | .543 |
| Table and chair | 1.000 | .557 |
| Wash mechine | 1.000 | .914 |
| Bed | 1.000 | .907 |
| Gas cooker | 1.000 | .421 |
| Bank account | 1.000 | .421 |
| Extraction Method: Principal Component Analysis. | | |

| **Total Variance Explained** | | | | | | | | | |
| --- | --- | --- | --- | --- | --- | --- | --- | --- | --- |
| Component | Initial Eigenvalues | | | Extraction Sums of Squared Loadings | | | Rotation Sums of Squared Loadings | | |
|  | Total | % of Variance | Cumulative % | Total | % of Variance | Cumulative % | Total | % of Variance | Cumulative % |
| 1 | 3.365 | 30.595 | 30.595 | 3.365 | 30.595 | 30.595 | 2.313 | 21.030 | 21.030 |
| 2 | 1.603 | 14.576 | 45.172 | 1.603 | 14.576 | 45.172 | 1.922 | 17.473 | 38.504 |
| 3 | 1.119 | 10.170 | 55.342 | 1.119 | 10.170 | 55.342 | 1.709 | 15.538 | 54.041 |
| 4 | 1.018 | 9.258 | 64.600 | 1.018 | 9.258 | 64.600 | 1.161 | 10.558 | 64.600 |
| 5 | .893 | 8.119 | 72.719 |  |  |  |  |  |  |
| 6 | .847 | 7.699 | 80.417 |  |  |  |  |  |  |
| 7 | .718 | 6.526 | 86.943 |  |  |  |  |  |  |
| 8 | .540 | 4.907 | 91.850 |  |  |  |  |  |  |
| 9 | .427 | 3.879 | 95.729 |  |  |  |  |  |  |
| 10 | .351 | 3.195 | 98.924 |  |  |  |  |  |  |
| 11 | .118 | 1.076 | 100.000 |  |  |  |  |  |  |
| Extraction Method: Principal Component Analysis. | | | | | | | | | |

| **Component Matrix^a^** | | | | |
| --- | --- | --- | --- | --- |
|  | Component | | | |
|  | 1 | 2 | 3 | 4 |
| Radio | .736 |  | -.365 |  |
| Car | .711 | -.315 | -.302 |  |
| Refrigerator | .644 |  | -.371 |  |
| Table and chair | .626 |  | .331 |  |
| Computer | .545 |  |  | .369 |
| Bicycle | .462 |  |  | -.367 |
| Bank account | .405 |  | .391 |  |
| Bed | .581 | .734 |  |  |
| Wash mechine | .581 | .733 |  |  |
| Gas cooker | .391 |  | .450 |  |
| Phone |  |  | -.432 | .718 |
| Extraction Method: Principal Component Analysis. | | | | |
| a. 4 components extracted. | | | | |

| **Rotated Component Matrix^a^** | | | | |
| --- | --- | --- | --- | --- |
|  | Component | | | |
|  | 1 | 2 | 3 | 4 |
| Radio | .834 |  |  |  |
| Car | .808 |  |  |  |
| Refrigerator | .785 |  |  |  |
| Wash mechine |  | .938 |  |  |
| Bed |  | .934 |  |  |
| Table and chair |  |  | .672 |  |
| Gas cooker |  |  | .638 |  |
| Computer | .386 |  | .620 |  |
| Bank account |  |  | .575 |  |
| Phone |  |  |  | -.890 |
| Bicycle |  |  |  | .565 |
| Extraction Method: Principal Component Analysis.  Rotation Method: Varimax with Kaiser Normalization.^a^ | | | | |
| a. Rotation converged in 5 iterations. | | | | |

| **Component Transformation Matrix** | | | | |
| --- | --- | --- | --- | --- |
| Component | 1 | 2 | 3 | 4 |
| 1 | .702 | .450 | .524 | .175 |
| 2 | -.425 | .855 | -.069 | -.289 |
| 3 | -.571 | -.069 | .667 | .474 |
| 4 | -.031 | -.247 | .526 | -.813 |
| Extraction Method: Principal Component Analysis.  Rotation Method: Varimax with Kaiser Normalization. | | | | |

FACTOR

/VARIABLES W2WorkiRadio W3Refrigertor W4Car W12Phone W5Bicycle W7Computer W9Table W13Wmechine

W10Bed W6BankAccunt

/MISSING LISTWISE

/ANALYSIS W2WorkiRadio W3Refrigertor W4Car W12Phone W5Bicycle W7Computer W9Table W13Wmechine

W10Bed W6BankAccunt

/PRINT UNIVARIATE INITIAL CORRELATION KMO AIC EXTRACTION ROTATION

/FORMAT SORT BLANK(.30)

/CRITERIA MINEIGEN(1) ITERATE(25)

/EXTRACTION PC

/CRITERIA ITERATE(25)

/ROTATION VARIMAX

/METHOD=CORRELATION.

**Factor Analysis**

| **Notes** | | |
| --- | --- | --- |
| Output Created | | 05-FEB-2022 08:51:55 |
| Comments | |  |
| Input | Data | C:\Users\Admin\Documents\Sagal thesis SPSS data Analysis UPDATED\sagal MSc thesis DATA.sav |
|  | Active Dataset | DataSet1 |
|  | File Label | EPIDATA FI THESIS by SAGAL'S MSc 630 DATA |
|  | Filter | <none> |
|  | Weight | <none> |
|  | Split File | <none> |
|  | N of Rows in Working Data File | 630 |
| Missing Value Handling | Definition of Missing | MISSING=EXCLUDE: User-defined missing values are treated as missing. |
|  | Cases Used | LISTWISE: Statistics are based on cases with no missing values for any variable used. |
| Syntax | | FACTOR  /VARIABLES W2WorkiRadio W3Refrigertor W4Car W12Phone W5Bicycle W7Computer W9Table W13Wmechine  W10Bed W6BankAccunt  /MISSING LISTWISE  /ANALYSIS W2WorkiRadio W3Refrigertor W4Car W12Phone W5Bicycle W7Computer W9Table W13Wmechine  W10Bed W6BankAccunt  /PRINT UNIVARIATE INITIAL CORRELATION KMO AIC EXTRACTION ROTATION  /FORMAT SORT BLANK(.30)  /PLOT EIGEN  /CRITERIA MINEIGEN(1) ITERATE(25)  /EXTRACTION PC  /CRITERIA ITERATE(25)  /ROTATION VARIMAX  /METHOD=CORRELATION. |
| Resources | Processor Time | 00:00:00.22 |
|  | Elapsed Time | 00:00:00.23 |
|  | Maximum Memory Required | 13688 (13.367K) bytes |

| **Descriptive Statistics** | | | |
| --- | --- | --- | --- |
|  | Mean | Std. Deviation | Analysis N |
| Radio | .26 | .440 | 630 |
| Refrigerator | .50 | .500 | 630 |
| Car | .21 | .407 | 630 |
| Phone | .99 | .079 | 630 |
| Bicycle | .18 | .385 | 630 |
| Computer | .52 | .500 | 630 |
| Table and chair | .52 | .500 | 630 |
| Wash mechine | .76 | .426 | 630 |
| Bed | .81 | .393 | 630 |
| Bank account | .40 | .490 | 630 |

| **Correlation Matrix** | | | | | | | | | | | |
| --- | --- | --- | --- | --- | --- | --- | --- | --- | --- | --- | --- |
|  | | Radio | Refrigerator | Car | Phone | Bicycle | Computer | Table and chair | Wash mechine | Bed | Bank account |
| Correlation | Radio | 1.000 | .596 | .598 | -.043 | .245 | .348 | .348 | .265 | .215 | .196 |
|  | Refrigerator | .596 | 1.000 | .515 | -.080 | .148 | .235 | .261 | .164 | .202 | .130 |
|  | Car | .598 | .515 | 1.000 | -.057 | .366 | .338 | .307 | .233 | .190 | .109 |
|  | Phone | -.043 | -.080 | -.057 | 1.000 | -.170 | .003 | -.077 | .049 | .063 | -.017 |
|  | Bicycle | .245 | .148 | .366 | -.170 | 1.000 | .253 | .236 | .108 | .165 | .108 |
|  | Computer | .348 | .235 | .338 | .003 | .253 | 1.000 | .402 | .105 | .117 | .129 |
|  | Table and chair | .348 | .261 | .307 | -.077 | .236 | .402 | 1.000 | .254 | .247 | .298 |
|  | Wash mechine | .265 | .164 | .233 | .049 | .108 | .105 | .254 | 1.000 | .868 | .240 |
|  | Bed | .215 | .202 | .190 | .063 | .165 | .117 | .247 | .868 | 1.000 | .245 |
|  | Bank account | .196 | .130 | .109 | -.017 | .108 | .129 | .298 | .240 | .245 | 1.000 |

| **KMO and Bartlett's Test** | | |
| --- | --- | --- |
| Kaiser-Meyer-Olkin Measure of Sampling Adequacy. | | .688 |
| Bartlett's Test of Sphericity | Approx. Chi-Square | 2064.995 |
|  | df | 45 |
|  | Sig. | .000 |

| **Anti-image Matrices** | | | | | | | | | | | |
| --- | --- | --- | --- | --- | --- | --- | --- | --- | --- | --- | --- |
|  | | Radio | Refrigerator | Car | Phone | Bicycle | Computer | Table and chair | Wash mechine | Bed | Bank account |
| Anti-image Covariance | Radio | .485 | -.218 | -.162 | -.015 | -.026 | -.080 | -.050 | -.053 | .040 | -.054 |
|  | Refrigerator | -.218 | .575 | -.148 | .061 | .080 | .009 | -.026 | .065 | -.072 | -.003 |
|  | Car | -.162 | -.148 | .531 | -.017 | -.178 | -.070 | -.025 | -.048 | .037 | .041 |
|  | Phone | -.015 | .061 | -.017 | .948 | .153 | -.057 | .056 | .014 | -.039 | .004 |
|  | Bicycle | -.026 | .080 | -.178 | .153 | .784 | -.084 | -.054 | .063 | -.076 | -.022 |
|  | Computer | -.080 | .009 | -.070 | -.057 | -.084 | .758 | -.215 | .024 | -.013 | .004 |
|  | Table and chair | -.050 | -.026 | -.025 | .056 | -.054 | -.215 | .713 | -.023 | -.007 | -.165 |
|  | Wash mechine | -.053 | .065 | -.048 | .014 | .063 | .024 | -.023 | .226 | -.195 | -.015 |
|  | Bed | .040 | -.072 | .037 | -.039 | -.076 | -.013 | -.007 | -.195 | .228 | -.027 |
|  | Bank account | -.054 | -.003 | .041 | .004 | -.022 | .004 | -.165 | -.015 | -.027 | .870 |
| Anti-image Correlation | Radio | .771^a^ | -.414 | -.320 | -.022 | -.042 | -.132 | -.086 | -.161 | .120 | -.082 |
|  | Refrigerator | -.414 | .717^a^ | -.268 | .083 | .119 | .013 | -.041 | .181 | -.198 | -.005 |
|  | Car | -.320 | -.268 | .782^a^ | -.024 | -.275 | -.111 | -.040 | -.138 | .107 | .061 |
|  | Phone | -.022 | .083 | -.024 | .486^a^ | .177 | -.067 | .068 | .030 | -.083 | .004 |
|  | Bicycle | -.042 | .119 | -.275 | .177 | .680^a^ | -.109 | -.073 | .150 | -.179 | -.026 |
|  | Computer | -.132 | .013 | -.111 | -.067 | -.109 | .803^a^ | -.292 | .059 | -.032 | .005 |
|  | Table and chair | -.086 | -.041 | -.040 | .068 | -.073 | -.292 | .824^a^ | -.058 | -.017 | -.209 |
|  | Wash mechine | -.161 | .181 | -.138 | .030 | .150 | .059 | -.058 | .554^a^ | -.859 | -.033 |
|  | Bed | .120 | -.198 | .107 | -.083 | -.179 | -.032 | -.017 | -.859 | .552^a^ | -.060 |
|  | Bank account | -.082 | -.005 | .061 | .004 | -.026 | .005 | -.209 | -.033 | -.060 | .835^a^ |
| a. Measures of Sampling Adequacy(MSA) | | | | | | | | | | | |

| **Communalities** | | |
| --- | --- | --- |
|  | Initial | Extraction |
| Radio | 1.000 | .726 |
| Refrigerator | 1.000 | .640 |
| Car | 1.000 | .674 |
| Phone | 1.000 | .504 |
| Bicycle | 1.000 | .458 |
| Computer | 1.000 | .372 |
| Table and chair | 1.000 | .473 |
| Wash mechine | 1.000 | .877 |
| Bed | 1.000 | .876 |
| Bank account | 1.000 | .329 |
| Extraction Method: Principal Component Analysis. | | |

| **Total Variance Explained** | | | | | | | | | |
| --- | --- | --- | --- | --- | --- | --- | --- | --- | --- |
| Component | Initial Eigenvalues | | | Extraction Sums of Squared Loadings | | | Rotation Sums of Squared Loadings | | |
|  | Total | % of Variance | Cumulative % | Total | % of Variance | Cumulative % | Total | % of Variance | Cumulative % |
| 1 | 3.253 | 32.532 | 32.532 | 3.253 | 32.532 | 32.532 | 2.482 | 24.815 | 24.815 |
| 2 | 1.600 | 16.002 | 48.533 | 1.600 | 16.002 | 48.533 | 2.085 | 20.853 | 45.668 |
| 3 | 1.074 | 10.738 | 59.272 | 1.074 | 10.738 | 59.272 | 1.360 | 13.604 | 59.272 |
| 4 | 1.000 | 9.995 | 69.267 |  |  |  |  |  |  |
| 5 | .884 | 8.836 | 78.104 |  |  |  |  |  |  |
| 6 | .730 | 7.304 | 85.407 |  |  |  |  |  |  |
| 7 | .541 | 5.408 | 90.815 |  |  |  |  |  |  |
| 8 | .434 | 4.343 | 95.158 |  |  |  |  |  |  |
| 9 | .366 | 3.657 | 98.815 |  |  |  |  |  |  |
| 10 | .119 | 1.185 | 100.000 |  |  |  |  |  |  |
| Extraction Method: Principal Component Analysis. | | | | | | | | | |

| **Component Matrix^a^** | | | |
| --- | --- | --- | --- |
|  | Component | | |
|  | 1 | 2 | 3 |
| Radio | .753 |  |  |
| Car | .719 | -.332 |  |
| Refrigerator | .644 |  | .372 |
| Table and chair | .620 |  |  |
| Computer | .535 |  |  |
| Bicycle | .463 |  | -.432 |
| Bank account | .400 |  | -.337 |
| Bed | .590 | .726 |  |
| Wash mechine | .595 | .723 |  |
| Phone |  |  | .641 |
| Extraction Method: Principal Component Analysis. | | | |
| a. 3 components extracted. | | | |

| **Rotated Component Matrix^a^** | | | |
| --- | --- | --- | --- |
|  | Component | | |
|  | 1 | 2 | 3 |
| Radio | .835 |  |  |
| Car | .802 |  |  |
| Refrigerator | .797 |  |  |
| Computer | .499 |  | .343 |
| Bed |  | .928 |  |
| Wash mechine |  | .925 |  |
| Bank account |  | .444 | .359 |
| Phone |  |  | -.690 |
| Bicycle |  |  | .611 |
| Table and chair | .373 | .319 | .482 |
| Extraction Method: Principal Component Analysis.  Rotation Method: Varimax with Kaiser Normalization.^a^ | | | |
| a. Rotation converged in 4 iterations. | | | |

| **Component Transformation Matrix** | | | |
| --- | --- | --- | --- |
| Component | 1 | 2 | 3 |
| 1 | .772 | .544 | .329 |
| 2 | -.456 | .834 | -.311 |
| 3 | .443 | -.090 | -.892 |
| Extraction Method: Principal Component Analysis.  Rotation Method: Varimax with Kaiser Normalization. | | | |

FACTOR

/VARIABLES W2WorkiRadio W3Refrigertor W4Car W5Bicycle W7Computer W9Table W13Wmechine W10Bed

W6BankAccunt

/MISSING LISTWISE

/ANALYSIS W2WorkiRadio W3Refrigertor W4Car W5Bicycle W7Computer W9Table W13Wmechine W10Bed

W6BankAccunt

/PRINT UNIVARIATE INITIAL CORRELATION KMO AIC EXTRACTION ROTATION

/FORMAT SORT BLANK(.30)

/CRITERIA MINEIGEN(1) ITERATE(25)

/EXTRACTION PC

/CRITERIA ITERATE(25)

/ROTATION VARIMAX

/METHOD=CORRELATION.

**Factor Analysis**

| **Notes** | | |
| --- | --- | --- |
| Output Created | | 05-FEB-2022 08:52:22 |
| Comments | |  |
| Input | Data | C:\Users\Admin\Documents\Sagal thesis SPSS data Analysis UPDATED\sagal MSc thesis DATA.sav |
|  | Active Dataset | DataSet1 |
|  | File Label | EPIDATA FI THESIS by SAGAL'S MSc 630 DATA |
|  | Filter | <none> |
|  | Weight | <none> |
|  | Split File | <none> |
|  | N of Rows in Working Data File | 630 |
| Missing Value Handling | Definition of Missing | MISSING=EXCLUDE: User-defined missing values are treated as missing. |
|  | Cases Used | LISTWISE: Statistics are based on cases with no missing values for any variable used. |
| Syntax | | FACTOR  /VARIABLES W2WorkiRadio W3Refrigertor W4Car W5Bicycle W7Computer W9Table W13Wmechine W10Bed  W6BankAccunt  /MISSING LISTWISE  /ANALYSIS W2WorkiRadio W3Refrigertor W4Car W5Bicycle W7Computer W9Table W13Wmechine W10Bed  W6BankAccunt  /PRINT UNIVARIATE INITIAL CORRELATION KMO AIC EXTRACTION ROTATION  /FORMAT SORT BLANK(.30)  /PLOT EIGEN  /CRITERIA MINEIGEN(1) ITERATE(25)  /EXTRACTION PC  /CRITERIA ITERATE(25)  /ROTATION VARIMAX  /METHOD=CORRELATION. |
| Resources | Processor Time | 00:00:00.37 |
|  | Elapsed Time | 00:00:00.25 |
|  | Maximum Memory Required | 11368 (11.102K) bytes |

| **Descriptive Statistics** | | | |
| --- | --- | --- | --- |
|  | Mean | Std. Deviation | Analysis N |
| Radio | .26 | .440 | 630 |
| Refrigerator | .50 | .500 | 630 |
| Car | .21 | .407 | 630 |
| Bicycle | .18 | .385 | 630 |
| Computer | .52 | .500 | 630 |
| Table and chair | .52 | .500 | 630 |
| Wash mechine | .76 | .426 | 630 |
| Bed | .81 | .393 | 630 |
| Bank account | .40 | .490 | 630 |

| **Correlation Matrix** | | | | | | | | | | |
| --- | --- | --- | --- | --- | --- | --- | --- | --- | --- | --- |
|  | | Radio | Refrigerator | Car | Bicycle | Computer | Table and chair | Wash mechine | Bed | Bank account |
| Correlation | Radio | 1.000 | .596 | .598 | .245 | .348 | .348 | .265 | .215 | .196 |
|  | Refrigerator | .596 | 1.000 | .515 | .148 | .235 | .261 | .164 | .202 | .130 |
|  | Car | .598 | .515 | 1.000 | .366 | .338 | .307 | .233 | .190 | .109 |
|  | Bicycle | .245 | .148 | .366 | 1.000 | .253 | .236 | .108 | .165 | .108 |
|  | Computer | .348 | .235 | .338 | .253 | 1.000 | .402 | .105 | .117 | .129 |
|  | Table and chair | .348 | .261 | .307 | .236 | .402 | 1.000 | .254 | .247 | .298 |
|  | Wash mechine | .265 | .164 | .233 | .108 | .105 | .254 | 1.000 | .868 | .240 |
|  | Bed | .215 | .202 | .190 | .165 | .117 | .247 | .868 | 1.000 | .245 |
|  | Bank account | .196 | .130 | .109 | .108 | .129 | .298 | .240 | .245 | 1.000 |

| **KMO and Bartlett's Test** | | |
| --- | --- | --- |
| Kaiser-Meyer-Olkin Measure of Sampling Adequacy. | | .694 |
| Bartlett's Test of Sphericity | Approx. Chi-Square | 2032.444 |
|  | df | 36 |
|  | Sig. | .000 |

| **Anti-image Matrices** | | | | | | | | | | |
| --- | --- | --- | --- | --- | --- | --- | --- | --- | --- | --- |
|  | | Radio | Refrigerator | Car | Bicycle | Computer | Table and chair | Wash mechine | Bed | Bank account |
| Anti-image Covariance | Radio | .485 | -.219 | -.163 | -.024 | -.082 | -.050 | -.053 | .040 | -.054 |
|  | Refrigerator | -.219 | .579 | -.148 | .073 | .012 | -.030 | .065 | -.070 | -.004 |
|  | Car | -.163 | -.148 | .531 | -.181 | -.072 | -.024 | -.048 | .037 | .041 |
|  | Bicycle | -.024 | .073 | -.181 | .810 | -.078 | -.066 | .063 | -.072 | -.023 |
|  | Computer | -.082 | .012 | -.072 | -.078 | .761 | -.214 | .025 | -.016 | .004 |
|  | Table and chair | -.050 | -.030 | -.024 | -.066 | -.214 | .716 | -.024 | -.004 | -.166 |
|  | Wash mechine | -.053 | .065 | -.048 | .063 | .025 | -.024 | .227 | -.196 | -.015 |
|  | Bed | .040 | -.070 | .037 | -.072 | -.016 | -.004 | -.196 | .230 | -.027 |
|  | Bank account | -.054 | -.004 | .041 | -.023 | .004 | -.166 | -.015 | -.027 | .870 |
| Anti-image Correlation | Radio | .771^a^ | -.413 | -.320 | -.039 | -.134 | -.084 | -.160 | .119 | -.082 |
|  | Refrigerator | -.413 | .723^a^ | -.267 | .106 | .019 | -.047 | .179 | -.192 | -.005 |
|  | Car | -.320 | -.267 | .782^a^ | -.275 | -.113 | -.038 | -.138 | .106 | .061 |
|  | Bicycle | -.039 | .106 | -.275 | .712^a^ | -.099 | -.086 | .147 | -.168 | -.027 |
|  | Computer | -.134 | .019 | -.113 | -.099 | .811^a^ | -.289 | .061 | -.037 | .005 |
|  | Table and chair | -.084 | -.047 | -.038 | -.086 | -.289 | .827^a^ | -.060 | -.011 | -.210 |
|  | Wash mechine | -.160 | .179 | -.138 | .147 | .061 | -.060 | .554^a^ | -.859 | -.033 |
|  | Bed | .119 | -.192 | .106 | -.168 | -.037 | -.011 | -.859 | .554^a^ | -.060 |
|  | Bank account | -.082 | -.005 | .061 | -.027 | .005 | -.210 | -.033 | -.060 | .834^a^ |
| a. Measures of Sampling Adequacy(MSA) | | | | | | | | | | |

| **Communalities** | | |
| --- | --- | --- |
|  | Initial | Extraction |
| Radio | 1.000 | .725 |
| Refrigerator | 1.000 | .691 |
| Car | 1.000 | .699 |
| Bicycle | 1.000 | .320 |
| Computer | 1.000 | .541 |
| Table and chair | 1.000 | .609 |
| Wash mechine | 1.000 | .912 |
| Bed | 1.000 | .903 |
| Bank account | 1.000 | .450 |
| Extraction Method: Principal Component Analysis. | | |

| **Total Variance Explained** | | | | | | | | | |
| --- | --- | --- | --- | --- | --- | --- | --- | --- | --- |
| Component | Initial Eigenvalues | | | Extraction Sums of Squared Loadings | | | Rotation Sums of Squared Loadings | | |
|  | Total | % of Variance | Cumulative % | Total | % of Variance | Cumulative % | Total | % of Variance | Cumulative % |
| 1 | 3.248 | 36.092 | 36.092 | 3.248 | 36.092 | 36.092 | 2.181 | 24.236 | 24.236 |
| 2 | 1.567 | 17.417 | 53.508 | 1.567 | 17.417 | 53.508 | 1.930 | 21.440 | 45.676 |
| 3 | 1.034 | 11.494 | 65.002 | 1.034 | 11.494 | 65.002 | 1.739 | 19.326 | 65.002 |
| 4 | .902 | 10.027 | 75.029 |  |  |  |  |  |  |
| 5 | .770 | 8.561 | 83.590 |  |  |  |  |  |  |
| 6 | .553 | 6.150 | 89.739 |  |  |  |  |  |  |
| 7 | .439 | 4.874 | 94.613 |  |  |  |  |  |  |
| 8 | .366 | 4.063 | 98.676 |  |  |  |  |  |  |
| 9 | .119 | 1.324 | 100.000 |  |  |  |  |  |  |
| Extraction Method: Principal Component Analysis. | | | | | | | | | |

| **Component Matrix^a^** | | | |
| --- | --- | --- | --- |
|  | Component | | |
|  | 1 | 2 | 3 |
| Radio | .752 | -.302 |  |
| Car | .718 | -.351 |  |
| Refrigerator | .642 | -.313 | -.425 |
| Table and chair | .619 |  | .472 |
| Computer | .536 | -.307 | .400 |
| Bicycle | .458 |  |  |
| Bed | .595 | .729 |  |
| Wash mechine | .599 | .726 |  |
| Bank account | .401 |  | .479 |
| Extraction Method: Principal Component Analysis. | | | |
| a. 3 components extracted. | | | |

| **Rotated Component Matrix^a^** | | | |
| --- | --- | --- | --- |
|  | Component | | |
|  | 1 | 2 | 3 |
| Refrigerator | .823 |  |  |
| Radio | .800 |  |  |
| Car | .792 |  |  |
| Wash mechine |  | .939 |  |
| Bed |  | .935 |  |
| Table and chair |  |  | .739 |
| Computer |  |  | .672 |
| Bank account |  | .322 | .578 |
| Bicycle |  |  | .502 |
| Extraction Method: Principal Component Analysis.  Rotation Method: Varimax with Kaiser Normalization.^a^ | | | |
| a. Rotation converged in 5 iterations. | | | |

| **Component Transformation Matrix** | | | |
| --- | --- | --- | --- |
| Component | 1 | 2 | 3 |
| 1 | .680 | .473 | .559 |
| 2 | -.479 | .865 | -.150 |
| 3 | -.555 | -.166 | .815 |
| Extraction Method: Principal Component Analysis.  Rotation Method: Varimax with Kaiser Normalization. | | | |

FACTOR

/VARIABLES W2WorkiRadio W3Refrigertor W4Car W7Computer W9Table W13Wmechine W10Bed W6BankAccunt

/MISSING LISTWISE

/ANALYSIS W2WorkiRadio W3Refrigertor W4Car W7Computer W9Table W13Wmechine W10Bed W6BankAccunt

/PRINT UNIVARIATE INITIAL CORRELATION KMO AIC EXTRACTION ROTATION

/FORMAT SORT BLANK(.30)

/CRITERIA MINEIGEN(1) ITERATE(25)

/EXTRACTION PC

/CRITERIA ITERATE(25)

/ROTATION VARIMAX

/METHOD=CORRELATION.

**Factor Analysis**

| **Notes** | | |
| --- | --- | --- |
| Output Created | | 05-FEB-2022 08:52:47 |
| Comments | |  |
| Input | Data | C:\Users\Admin\Documents\Sagal thesis SPSS data Analysis UPDATED\sagal MSc thesis DATA.sav |
|  | Active Dataset | DataSet1 |
|  | File Label | EPIDATA FI THESIS by SAGAL'S MSc 630 DATA |
|  | Filter | <none> |
|  | Weight | <none> |
|  | Split File | <none> |
|  | N of Rows in Working Data File | 630 |
| Missing Value Handling | Definition of Missing | MISSING=EXCLUDE: User-defined missing values are treated as missing. |
|  | Cases Used | LISTWISE: Statistics are based on cases with no missing values for any variable used. |
| Syntax | | FACTOR  /VARIABLES W2WorkiRadio W3Refrigertor W4Car W7Computer W9Table W13Wmechine W10Bed W6BankAccunt  /MISSING LISTWISE  /ANALYSIS W2WorkiRadio W3Refrigertor W4Car W7Computer W9Table W13Wmechine W10Bed W6BankAccunt  /PRINT UNIVARIATE INITIAL CORRELATION KMO AIC EXTRACTION ROTATION  /FORMAT SORT BLANK(.30)  /PLOT EIGEN  /CRITERIA MINEIGEN(1) ITERATE(25)  /EXTRACTION PC  /CRITERIA ITERATE(25)  /ROTATION VARIMAX  /METHOD=CORRELATION. |
| Resources | Processor Time | 00:00:00.27 |
|  | Elapsed Time | 00:00:00.23 |
|  | Maximum Memory Required | 9264 (9.047K) bytes |

| **Descriptive Statistics** | | | |
| --- | --- | --- | --- |
|  | Mean | Std. Deviation | Analysis N |
| Radio | .26 | .440 | 630 |
| Refrigerator | .50 | .500 | 630 |
| Car | .21 | .407 | 630 |
| Computer | .52 | .500 | 630 |
| Table and chair | .52 | .500 | 630 |
| Wash mechine | .76 | .426 | 630 |
| Bed | .81 | .393 | 630 |
| Bank account | .40 | .490 | 630 |

| **Correlation Matrix** | | | | | | | | | |
| --- | --- | --- | --- | --- | --- | --- | --- | --- | --- |
|  | | Radio | Refrigerator | Car | Computer | Table and chair | Wash mechine | Bed | Bank account |
| Correlation | Radio | 1.000 | .596 | .598 | .348 | .348 | .265 | .215 | .196 |
|  | Refrigerator | .596 | 1.000 | .515 | .235 | .261 | .164 | .202 | .130 |
|  | Car | .598 | .515 | 1.000 | .338 | .307 | .233 | .190 | .109 |
|  | Computer | .348 | .235 | .338 | 1.000 | .402 | .105 | .117 | .129 |
|  | Table and chair | .348 | .261 | .307 | .402 | 1.000 | .254 | .247 | .298 |
|  | Wash mechine | .265 | .164 | .233 | .105 | .254 | 1.000 | .868 | .240 |
|  | Bed | .215 | .202 | .190 | .117 | .247 | .868 | 1.000 | .245 |
|  | Bank account | .196 | .130 | .109 | .129 | .298 | .240 | .245 | 1.000 |

| **KMO and Bartlett's Test** | | |
| --- | --- | --- |
| Kaiser-Meyer-Olkin Measure of Sampling Adequacy. | | .693 |
| Bartlett's Test of Sphericity | Approx. Chi-Square | 1901.453 |
|  | df | 28 |
|  | Sig. | .000 |

| **Anti-image Matrices** | | | | | | | | | |
| --- | --- | --- | --- | --- | --- | --- | --- | --- | --- |
|  | | Radio | Refrigerator | Car | Computer | Table and chair | Wash mechine | Bed | Bank account |
| Anti-image Covariance | Radio | .486 | -.220 | -.182 | -.085 | -.052 | -.053 | .039 | -.054 |
|  | Refrigerator | -.220 | .585 | -.144 | .020 | -.025 | .061 | -.066 | -.002 |
|  | Car | -.182 | -.144 | .575 | -.098 | -.042 | -.037 | .023 | .039 |
|  | Computer | -.085 | .020 | -.098 | .769 | -.224 | .032 | -.023 | .002 |
|  | Table and chair | -.052 | -.025 | -.042 | -.224 | .722 | -.020 | -.011 | -.169 |
|  | Wash mechine | -.053 | .061 | -.037 | .032 | -.020 | .232 | -.200 | -.013 |
|  | Bed | .039 | -.066 | .023 | -.023 | -.011 | -.200 | .236 | -.030 |
|  | Bank account | -.054 | -.002 | .039 | .002 | -.169 | -.013 | -.030 | .871 |
| Anti-image Correlation | Radio | .755^a^ | -.412 | -.345 | -.139 | -.088 | -.157 | .114 | -.084 |
|  | Refrigerator | -.412 | .739^a^ | -.248 | .030 | -.038 | .166 | -.178 | -.002 |
|  | Car | -.345 | -.248 | .807^a^ | -.147 | -.065 | -.102 | .063 | .055 |
|  | Computer | -.139 | .030 | -.147 | .778^a^ | -.300 | .077 | -.055 | .003 |
|  | Table and chair | -.088 | -.038 | -.065 | -.300 | .812^a^ | -.048 | -.026 | -.213 |
|  | Wash mechine | -.157 | .166 | -.102 | .077 | -.048 | .563^a^ | -.856 | -.030 |
|  | Bed | .114 | -.178 | .063 | -.055 | -.026 | -.856 | .561^a^ | -.065 |
|  | Bank account | -.084 | -.002 | .055 | .003 | -.213 | -.030 | -.065 | .827^a^ |
| a. Measures of Sampling Adequacy(MSA) | | | | | | | | | |

| **Communalities** | | |
| --- | --- | --- |
|  | Initial | Extraction |
| Radio | 1.000 | .733 |
| Refrigerator | 1.000 | .679 |
| Car | 1.000 | .691 |
| Computer | 1.000 | .541 |
| Table and chair | 1.000 | .651 |
| Wash mechine | 1.000 | .918 |
| Bed | 1.000 | .914 |
| Bank account | 1.000 | .535 |
| Extraction Method: Principal Component Analysis. | | |

| **Total Variance Explained** | | | | | | | | | |
| --- | --- | --- | --- | --- | --- | --- | --- | --- | --- |
| Component | Initial Eigenvalues | | | Extraction Sums of Squared Loadings | | | Rotation Sums of Squared Loadings | | |
|  | Total | % of Variance | Cumulative % | Total | % of Variance | Cumulative % | Total | % of Variance | Cumulative % |
| 1 | 3.094 | 38.676 | 38.676 | 3.094 | 38.676 | 38.676 | 2.229 | 27.859 | 27.859 |
| 2 | 1.547 | 19.343 | 58.019 | 1.547 | 19.343 | 58.019 | 1.895 | 23.683 | 51.542 |
| 3 | 1.021 | 12.768 | 70.787 | 1.021 | 12.768 | 70.787 | 1.540 | 19.245 | 70.787 |
| 4 | .816 | 10.196 | 80.983 |  |  |  |  |  |  |
| 5 | .555 | 6.938 | 87.921 |  |  |  |  |  |  |
| 6 | .473 | 5.907 | 93.828 |  |  |  |  |  |  |
| 7 | .370 | 4.628 | 98.456 |  |  |  |  |  |  |
| 8 | .123 | 1.544 | 100.000 |  |  |  |  |  |  |
| Extraction Method: Principal Component Analysis. | | | | | | | | | |

| **Component Matrix^a^** | | | |
| --- | --- | --- | --- |
|  | Component | | |
|  | 1 | 2 | 3 |
| Radio | .758 | -.348 |  |
| Car | .700 | -.370 |  |
| Refrigerator | .657 | -.369 | -.333 |
| Table and chair | .618 |  | .515 |
| Computer | .522 | -.317 | .409 |
| Bed | .616 | .712 |  |
| Wash mechine | .629 | .700 |  |
| Bank account | .412 |  | .560 |
| Extraction Method: Principal Component Analysis. | | | |
| a. 3 components extracted. | | | |

| **Rotated Component Matrix^a^** | | | |
| --- | --- | --- | --- |
|  | Component | | |
|  | 1 | 2 | 3 |
| Refrigerator | .816 |  |  |
| Radio | .815 |  |  |
| Car | .812 |  |  |
| Bed |  | .939 |  |
| Wash mechine |  | .939 |  |
| Table and chair |  |  | .757 |
| Bank account |  |  | .679 |
| Computer | .365 |  | .630 |
| Extraction Method: Principal Component Analysis.  Rotation Method: Varimax with Kaiser Normalization.^a^ | | | |
| a. Rotation converged in 5 iterations. | | | |

| **Component Transformation Matrix** | | | |
| --- | --- | --- | --- |
| Component | 1 | 2 | 3 |
| 1 | .712 | .494 | .499 |
| 2 | -.547 | .836 | -.047 |
| 3 | -.440 | -.240 | .865 |
| Extraction Method: Principal Component Analysis.  Rotation Method: Varimax with Kaiser Normalization. | | | |

FACTOR

/VARIABLES W2WorkiRadio W3Refrigertor W4Car W7Computer W9Table W13Wmechine W6BankAccunt

/MISSING LISTWISE

/ANALYSIS W2WorkiRadio W3Refrigertor W4Car W7Computer W9Table W13Wmechine W6BankAccunt

/PRINT UNIVARIATE INITIAL CORRELATION KMO AIC EXTRACTION ROTATION

/FORMAT SORT BLANK(.30)

/CRITERIA MINEIGEN(1) ITERATE(25)

/EXTRACTION PC

/CRITERIA ITERATE(25)

/ROTATION VARIMAX

/METHOD=CORRELATION.

**Factor Analysis**

| **Notes** | | |
| --- | --- | --- |
| Output Created | | 05-FEB-2022 08:53:35 |
| Comments | |  |
| Input | Data | C:\Users\Admin\Documents\Sagal thesis SPSS data Analysis UPDATED\sagal MSc thesis DATA.sav |
|  | Active Dataset | DataSet1 |
|  | File Label | EPIDATA FI THESIS by SAGAL'S MSc 630 DATA |
|  | Filter | <none> |
|  | Weight | <none> |
|  | Split File | <none> |
|  | N of Rows in Working Data File | 630 |
| Missing Value Handling | Definition of Missing | MISSING=EXCLUDE: User-defined missing values are treated as missing. |
|  | Cases Used | LISTWISE: Statistics are based on cases with no missing values for any variable used. |
| Syntax | | FACTOR  /VARIABLES W2WorkiRadio W3Refrigertor W4Car W7Computer W9Table W13Wmechine W6BankAccunt  /MISSING LISTWISE  /ANALYSIS W2WorkiRadio W3Refrigertor W4Car W7Computer W9Table W13Wmechine W6BankAccunt  /PRINT UNIVARIATE INITIAL CORRELATION KMO AIC EXTRACTION ROTATION  /FORMAT SORT BLANK(.30)  /PLOT EIGEN  /CRITERIA MINEIGEN(1) ITERATE(25)  /EXTRACTION PC  /CRITERIA ITERATE(25)  /ROTATION VARIMAX  /METHOD=CORRELATION. |
| Resources | Processor Time | 00:00:00.20 |
|  | Elapsed Time | 00:00:00.20 |
|  | Maximum Memory Required | 7376 (7.203K) bytes |

| **Descriptive Statistics** | | | |
| --- | --- | --- | --- |
|  | Mean | Std. Deviation | Analysis N |
| Radio | .26 | .440 | 630 |
| Refrigerator | .50 | .500 | 630 |
| Car | .21 | .407 | 630 |
| Computer | .52 | .500 | 630 |
| Table and chair | .52 | .500 | 630 |
| Wash mechine | .76 | .426 | 630 |
| Bank account | .40 | .490 | 630 |

| **Correlation Matrix** | | | | | | | | |
| --- | --- | --- | --- | --- | --- | --- | --- | --- |
|  | | Radio | Refrigerator | Car | Computer | Table and chair | Wash mechine | Bank account |
| Correlation | Radio | 1.000 | .596 | .598 | .348 | .348 | .265 | .196 |
|  | Refrigerator | .596 | 1.000 | .515 | .235 | .261 | .164 | .130 |
|  | Car | .598 | .515 | 1.000 | .338 | .307 | .233 | .109 |
|  | Computer | .348 | .235 | .338 | 1.000 | .402 | .105 | .129 |
|  | Table and chair | .348 | .261 | .307 | .402 | 1.000 | .254 | .298 |
|  | Wash mechine | .265 | .164 | .233 | .105 | .254 | 1.000 | .240 |
|  | Bank account | .196 | .130 | .109 | .129 | .298 | .240 | 1.000 |

| **KMO and Bartlett's Test** | | |
| --- | --- | --- |
| Kaiser-Meyer-Olkin Measure of Sampling Adequacy. | | .779 |
| Bartlett's Test of Sphericity | Approx. Chi-Square | 999.431 |
|  | df | 21 |
|  | Sig. | .000 |

| **Anti-image Matrices** | | | | | | | | |
| --- | --- | --- | --- | --- | --- | --- | --- | --- |
|  | | Radio | Refrigerator | Car | Computer | Table and chair | Wash mechine | Bank account |
| Anti-image Covariance | Radio | .493 | -.219 | -.189 | -.082 | -.051 | -.075 | -.050 |
|  | Refrigerator | -.219 | .604 | -.143 | .014 | -.029 | .019 | -.010 |
|  | Car | -.189 | -.143 | .577 | -.096 | -.041 | -.066 | .042 |
|  | Computer | -.082 | .014 | -.096 | .771 | -.226 | .047 | -.001 |
|  | Table and chair | -.051 | -.029 | -.041 | -.226 | .722 | -.108 | -.171 |
|  | Wash mechine | -.075 | .019 | -.066 | .047 | -.108 | .867 | -.145 |
|  | Bank account | -.050 | -.010 | .042 | -.001 | -.171 | -.145 | .875 |
| Anti-image Correlation | Radio | .763^a^ | -.401 | -.355 | -.134 | -.086 | -.115 | -.077 |
|  | Refrigerator | -.401 | .780^a^ | -.241 | .020 | -.044 | .027 | -.014 |
|  | Car | -.355 | -.241 | .802^a^ | -.144 | -.063 | -.094 | .060 |
|  | Computer | -.134 | .020 | -.144 | .782^a^ | -.302 | .057 | -.001 |
|  | Table and chair | -.086 | -.044 | -.063 | -.302 | .779^a^ | -.137 | -.216 |
|  | Wash mechine | -.115 | .027 | -.094 | .057 | -.137 | .798^a^ | -.166 |
|  | Bank account | -.077 | -.014 | .060 | -.001 | -.216 | -.166 | .733^a^ |
| a. Measures of Sampling Adequacy(MSA) | | | | | | | | |

| **Communalities** | | |
| --- | --- | --- |
|  | Initial | Extraction |
| Radio | 1.000 | .723 |
| Refrigerator | 1.000 | .647 |
| Car | 1.000 | .680 |
| Computer | 1.000 | .334 |
| Table and chair | 1.000 | .534 |
| Wash mechine | 1.000 | .413 |
| Bank account | 1.000 | .628 |
| Extraction Method: Principal Component Analysis. | | |

| **Total Variance Explained** | | | | | | | | | |
| --- | --- | --- | --- | --- | --- | --- | --- | --- | --- |
| Component | Initial Eigenvalues | | | Extraction Sums of Squared Loadings | | | Rotation Sums of Squared Loadings | | |
|  | Total | % of Variance | Cumulative % | Total | % of Variance | Cumulative % | Total | % of Variance | Cumulative % |
| 1 | 2.832 | 40.454 | 40.454 | 2.832 | 40.454 | 40.454 | 2.386 | 34.091 | 34.091 |
| 2 | 1.128 | 16.108 | 56.562 | 1.128 | 16.108 | 56.562 | 1.573 | 22.472 | 56.562 |
| 3 | .918 | 13.118 | 69.680 |  |  |  |  |  |  |
| 4 | .745 | 10.645 | 80.325 |  |  |  |  |  |  |
| 5 | .553 | 7.901 | 88.226 |  |  |  |  |  |  |
| 6 | .459 | 6.561 | 94.787 |  |  |  |  |  |  |
| 7 | .365 | 5.213 | 100.000 |  |  |  |  |  |  |
| Extraction Method: Principal Component Analysis. | | | | | | | | | |

| **Component Matrix^a^** | | |
| --- | --- | --- |
|  | Component | |
|  | 1 | 2 |
| Radio | .815 |  |
| Car | .759 | -.324 |
| Refrigerator | .710 | -.377 |
| Table and chair | .635 | .363 |
| Computer | .578 |  |
| Bank account | .385 | .693 |
| Wash mechine | .450 | .459 |
| Extraction Method: Principal Component Analysis. | | |
| a. 2 components extracted. | | |

| **Rotated Component Matrix^a^** | | |
| --- | --- | --- |
|  | Component | |
|  | 1 | 2 |
| Radio | .824 |  |
| Car | .817 |  |
| Refrigerator | .803 |  |
| Computer | .491 | .305 |
| Bank account |  | .792 |
| Table and chair | .360 | .636 |
| Wash mechine |  | .625 |
| Extraction Method: Principal Component Analysis.  Rotation Method: Varimax with Kaiser Normalization.^a^ | | |
| a. Rotation converged in 3 iterations. | | |

| **Component Transformation Matrix** | | |
| --- | --- | --- |
| Component | 1 | 2 |
| 1 | .859 | .511 |
| 2 | -.511 | .859 |
| Extraction Method: Principal Component Analysis.  Rotation Method: Varimax with Kaiser Normalization. | | |

FACTOR

/VARIABLES W2WorkiRadio W3Refrigertor W4Car W9Table W13Wmechine W6BankAccunt

/MISSING LISTWISE

/ANALYSIS W2WorkiRadio W3Refrigertor W4Car W9Table W13Wmechine W6BankAccunt

/PRINT UNIVARIATE INITIAL CORRELATION KMO AIC EXTRACTION ROTATION

/FORMAT SORT BLANK(.30)

/CRITERIA MINEIGEN(1) ITERATE(25)

/EXTRACTION PC

/CRITERIA ITERATE(25)

/ROTATION VARIMAX

/METHOD=CORRELATION.

**Factor Analysis**

| **Notes** | | |
| --- | --- | --- |
| Output Created | | 05-FEB-2022 08:53:58 |
| Comments | |  |
| Input | Data | C:\Users\Admin\Documents\Sagal thesis SPSS data Analysis UPDATED\sagal MSc thesis DATA.sav |
|  | Active Dataset | DataSet1 |
|  | File Label | EPIDATA FI THESIS by SAGAL'S MSc 630 DATA |
|  | Filter | <none> |
|  | Weight | <none> |
|  | Split File | <none> |
|  | N of Rows in Working Data File | 630 |
| Missing Value Handling | Definition of Missing | MISSING=EXCLUDE: User-defined missing values are treated as missing. |
|  | Cases Used | LISTWISE: Statistics are based on cases with no missing values for any variable used. |
| Syntax | | FACTOR  /VARIABLES W2WorkiRadio W3Refrigertor W4Car W9Table W13Wmechine W6BankAccunt  /MISSING LISTWISE  /ANALYSIS W2WorkiRadio W3Refrigertor W4Car W9Table W13Wmechine W6BankAccunt  /PRINT UNIVARIATE INITIAL CORRELATION KMO AIC EXTRACTION ROTATION  /FORMAT SORT BLANK(.30)  /PLOT EIGEN  /CRITERIA MINEIGEN(1) ITERATE(25)  /EXTRACTION PC  /CRITERIA ITERATE(25)  /ROTATION VARIMAX  /METHOD=CORRELATION. |
| Resources | Processor Time | 00:00:00.23 |
|  | Elapsed Time | 00:00:00.22 |
|  | Maximum Memory Required | 5704 (5.570K) bytes |

| **Descriptive Statistics** | | | |
| --- | --- | --- | --- |
|  | Mean | Std. Deviation | Analysis N |
| Radio | .26 | .440 | 630 |
| Refrigerator | .50 | .500 | 630 |
| Car | .21 | .407 | 630 |
| Table and chair | .52 | .500 | 630 |
| Wash mechine | .76 | .426 | 630 |
| Bank account | .40 | .490 | 630 |

| **Correlation Matrix** | | | | | | | |
| --- | --- | --- | --- | --- | --- | --- | --- |
|  | | Radio | Refrigerator | Car | Table and chair | Wash mechine | Bank account |
| Correlation | Radio | 1.000 | .596 | .598 | .348 | .265 | .196 |
|  | Refrigerator | .596 | 1.000 | .515 | .261 | .164 | .130 |
|  | Car | .598 | .515 | 1.000 | .307 | .233 | .109 |
|  | Table and chair | .348 | .261 | .307 | 1.000 | .254 | .298 |
|  | Wash mechine | .265 | .164 | .233 | .254 | 1.000 | .240 |
|  | Bank account | .196 | .130 | .109 | .298 | .240 | 1.000 |

| **KMO and Bartlett's Test** | | |
| --- | --- | --- |
| Kaiser-Meyer-Olkin Measure of Sampling Adequacy. | | .764 |
| Bartlett's Test of Sphericity | Approx. Chi-Square | 837.100 |
|  | df | 15 |
|  | Sig. | .000 |

| **Anti-image Matrices** | | | | | | | |
| --- | --- | --- | --- | --- | --- | --- | --- |
|  | | Radio | Refrigerator | Car | Table and chair | Wash mechine | Bank account |
| Anti-image Covariance | Radio | .502 | -.221 | -.207 | -.084 | -.071 | -.051 |
|  | Refrigerator | -.221 | .605 | -.144 | -.027 | .019 | -.010 |
|  | Car | -.207 | -.144 | .589 | -.078 | -.062 | .043 |
|  | Table and chair | -.084 | -.027 | -.078 | .795 | -.104 | -.189 |
|  | Wash mechine | -.071 | .019 | -.062 | -.104 | .870 | -.145 |
|  | Bank account | -.051 | -.010 | .043 | -.189 | -.145 | .875 |
| Anti-image Correlation | Radio | .734^a^ | -.402 | -.381 | -.133 | -.108 | -.078 |
|  | Refrigerator | -.402 | .767^a^ | -.241 | -.039 | .026 | -.014 |
|  | Car | -.381 | -.241 | .775^a^ | -.113 | -.087 | .060 |
|  | Table and chair | -.133 | -.039 | -.113 | .815^a^ | -.125 | -.227 |
|  | Wash mechine | -.108 | .026 | -.087 | -.125 | .812^a^ | -.166 |
|  | Bank account | -.078 | -.014 | .060 | -.227 | -.166 | .706^a^ |
| a. Measures of Sampling Adequacy(MSA) | | | | | | | |

| **Communalities** | | |
| --- | --- | --- |
|  | Initial | Extraction |
| Radio | 1.000 | .745 |
| Refrigerator | 1.000 | .689 |
| Car | 1.000 | .693 |
| Table and chair | 1.000 | .495 |
| Wash mechine | 1.000 | .450 |
| Bank account | 1.000 | .644 |
| Extraction Method: Principal Component Analysis. | | |

| **Total Variance Explained** | | | | | | | | | |
| --- | --- | --- | --- | --- | --- | --- | --- | --- | --- |
| Component | Initial Eigenvalues | | | Extraction Sums of Squared Loadings | | | Rotation Sums of Squared Loadings | | |
|  | Total | % of Variance | Cumulative % | Total | % of Variance | Cumulative % | Total | % of Variance | Cumulative % |
| 1 | 2.589 | 43.149 | 43.149 | 2.589 | 43.149 | 43.149 | 2.176 | 36.270 | 36.270 |
| 2 | 1.128 | 18.792 | 61.941 | 1.128 | 18.792 | 61.941 | 1.540 | 25.672 | 61.941 |
| 3 | .773 | 12.890 | 74.831 |  |  |  |  |  |  |
| 4 | .670 | 11.167 | 85.998 |  |  |  |  |  |  |
| 5 | .473 | 7.882 | 93.881 |  |  |  |  |  |  |
| 6 | .367 | 6.119 | 100.000 |  |  |  |  |  |  |
| Extraction Method: Principal Component Analysis. | | | | | | | | | |

| **Component Matrix^a^** | | |
| --- | --- | --- |
|  | Component | |
|  | 1 | 2 |
| Radio | .830 |  |
| Car | .768 | -.322 |
| Refrigerator | .742 | -.373 |
| Table and chair | .604 | .361 |
| Wash mechine | .485 | .464 |
| Bank account | .402 | .695 |
| Extraction Method: Principal Component Analysis. | | |
| a. 2 components extracted. | | |

| **Rotated Component Matrix^a^** | | |
| --- | --- | --- |
|  | Component | |
|  | 1 | 2 |
| Radio | .830 |  |
| Refrigerator | .827 |  |
| Car | .821 |  |
| Bank account |  | .802 |
| Wash mechine |  | .651 |
| Table and chair | .320 | .626 |
| Extraction Method: Principal Component Analysis.  Rotation Method: Varimax with Kaiser Normalization.^a^ | | |
| a. Rotation converged in 3 iterations. | | |

| **Component Transformation Matrix** | | |
| --- | --- | --- |
| Component | 1 | 2 |
| 1 | .847 | .531 |
| 2 | -.531 | .847 |
| Extraction Method: Principal Component Analysis.  Rotation Method: Varimax with Kaiser Normalization. | | |

FACTOR

/VARIABLES W2WorkiRadio W3Refrigertor W4Car W9Table W6BankAccunt

/MISSING LISTWISE

/ANALYSIS W2WorkiRadio W3Refrigertor W4Car W9Table W6BankAccunt

/PRINT UNIVARIATE INITIAL CORRELATION KMO AIC EXTRACTION ROTATION

/FORMAT SORT BLANK(.30)

/CRITERIA MINEIGEN(1) ITERATE(25)

/EXTRACTION PC

/CRITERIA ITERATE(25)

/ROTATION VARIMAX

/METHOD=CORRELATION.

**Factor Analysis**

| **Notes** | | |
| --- | --- | --- |
| Output Created | | 05-FEB-2022 08:54:32 |
| Comments | |  |
| Input | Data | C:\Users\Admin\Documents\Sagal thesis SPSS data Analysis UPDATED\sagal MSc thesis DATA.sav |
|  | Active Dataset | DataSet1 |
|  | File Label | EPIDATA FI THESIS by SAGAL'S MSc 630 DATA |
|  | Filter | <none> |
|  | Weight | <none> |
|  | Split File | <none> |
|  | N of Rows in Working Data File | 630 |
| Missing Value Handling | Definition of Missing | MISSING=EXCLUDE: User-defined missing values are treated as missing. |
|  | Cases Used | LISTWISE: Statistics are based on cases with no missing values for any variable used. |
| Syntax | | FACTOR  /VARIABLES W2WorkiRadio W3Refrigertor W4Car W9Table W6BankAccunt  /MISSING LISTWISE  /ANALYSIS W2WorkiRadio W3Refrigertor W4Car W9Table W6BankAccunt  /PRINT UNIVARIATE INITIAL CORRELATION KMO AIC EXTRACTION ROTATION  /FORMAT SORT BLANK(.30)  /PLOT EIGEN  /CRITERIA MINEIGEN(1) ITERATE(25)  /EXTRACTION PC  /CRITERIA ITERATE(25)  /ROTATION VARIMAX  /METHOD=CORRELATION. |
| Resources | Processor Time | 00:00:00.31 |
|  | Elapsed Time | 00:00:00.25 |
|  | Maximum Memory Required | 4248 (4.148K) bytes |

| **Descriptive Statistics** | | | |
| --- | --- | --- | --- |
|  | Mean | Std. Deviation | Analysis N |
| Radio | .26 | .440 | 630 |
| Refrigerator | .50 | .500 | 630 |
| Car | .21 | .407 | 630 |
| Table and chair | .52 | .500 | 630 |
| Bank account | .40 | .490 | 630 |

| **Correlation Matrix** | | | | | | |
| --- | --- | --- | --- | --- | --- | --- |
|  | | Radio | Refrigerator | Car | Table and chair | Bank account |
| Correlation | Radio | 1.000 | .596 | .598 | .348 | .196 |
|  | Refrigerator | .596 | 1.000 | .515 | .261 | .130 |
|  | Car | .598 | .515 | 1.000 | .307 | .109 |
|  | Table and chair | .348 | .261 | .307 | 1.000 | .298 |
|  | Bank account | .196 | .130 | .109 | .298 | 1.000 |

| **KMO and Bartlett's Test** | | |
| --- | --- | --- |
| Kaiser-Meyer-Olkin Measure of Sampling Adequacy. | | .743 |
| Bartlett's Test of Sphericity | Approx. Chi-Square | 749.990 |
|  | df | 10 |
|  | Sig. | .000 |

| **Anti-image Matrices** | | | | | | |
| --- | --- | --- | --- | --- | --- | --- |
|  | | Radio | Refrigerator | Car | Table and chair | Bank account |
| Anti-image Covariance | Radio | .507 | -.222 | -.216 | -.095 | -.066 |
|  | Refrigerator | -.222 | .605 | -.144 | -.025 | -.007 |
|  | Car | -.216 | -.144 | .594 | -.087 | .034 |
|  | Table and chair | -.095 | -.025 | -.087 | .807 | -.216 |
|  | Bank account | -.066 | -.007 | .034 | -.216 | .900 |
| Anti-image Correlation | Radio | .714^a^ | -.401 | -.394 | -.149 | -.098 |
|  | Refrigerator | -.401 | .762^a^ | -.240 | -.036 | -.010 |
|  | Car | -.394 | -.240 | .759^a^ | -.126 | .047 |
|  | Table and chair | -.149 | -.036 | -.126 | .782^a^ | -.253 |
|  | Bank account | -.098 | -.010 | .047 | -.253 | .673^a^ |
| a. Measures of Sampling Adequacy(MSA) | | | | | | |

| **Communalities** | | |
| --- | --- | --- |
|  | Initial | Extraction |
| Radio | 1.000 | .747 |
| Refrigerator | 1.000 | .683 |
| Car | 1.000 | .696 |
| Table and chair | 1.000 | .573 |
| Bank account | 1.000 | .784 |
| Extraction Method: Principal Component Analysis. | | |

| **Total Variance Explained** | | | | | | | | | |
| --- | --- | --- | --- | --- | --- | --- | --- | --- | --- |
| Component | Initial Eigenvalues | | | Extraction Sums of Squared Loadings | | | Rotation Sums of Squared Loadings | | |
|  | Total | % of Variance | Cumulative % | Total | % of Variance | Cumulative % | Total | % of Variance | Cumulative % |
| 1 | 2.432 | 48.635 | 48.635 | 2.432 | 48.635 | 48.635 | 2.171 | 43.418 | 43.418 |
| 2 | 1.051 | 21.021 | 69.655 | 1.051 | 21.021 | 69.655 | 1.312 | 26.238 | 69.655 |
| 3 | .670 | 13.402 | 83.057 |  |  |  |  |  |  |
| 4 | .479 | 9.574 | 92.631 |  |  |  |  |  |  |
| 5 | .368 | 7.369 | 100.000 |  |  |  |  |  |  |
| Extraction Method: Principal Component Analysis. | | | | | | | | | |

| **Component Matrix^a^** | | |
| --- | --- | --- |
|  | Component | |
|  | 1 | 2 |
| Radio | .847 |  |
| Car | .788 |  |
| Refrigerator | .777 |  |
| Table and chair | .593 | .471 |
| Bank account | .369 | .805 |
| Extraction Method: Principal Component Analysis. | | |
| a. 2 components extracted. | | |

| **Rotated Component Matrix^a^** | | |
| --- | --- | --- |
|  | Component | |
|  | 1 | 2 |
| Radio | .837 |  |
| Car | .829 |  |
| Refrigerator | .822 |  |
| Bank account |  | .885 |
| Table and chair | .329 | .682 |
| Extraction Method: Principal Component Analysis.  Rotation Method: Varimax with Kaiser Normalization.^a^ | | |
| a. Rotation converged in 3 iterations. | | |

| **Component Transformation Matrix** | | |
| --- | --- | --- |
| Component | 1 | 2 |
| 1 | .901 | .435 |
| 2 | -.435 | .901 |
| Extraction Method: Principal Component Analysis.  Rotation Method: Varimax with Kaiser Normalization. | | |

FACTOR

/VARIABLES W2WorkiRadio W3Refrigertor W4Car W6BankAccunt

/MISSING LISTWISE

/ANALYSIS W2WorkiRadio W3Refrigertor W4Car W6BankAccunt

/PRINT UNIVARIATE INITIAL CORRELATION KMO AIC EXTRACTION ROTATION

/FORMAT SORT BLANK(.30)

/PLOT EIGEN

/CRITERIA MINEIGEN(1) ITERATE(25)

/EXTRACTION PC

/CRITERIA ITERATE(25)

/ROTATION VARIMAX

/METHOD=CORRELATION.

**Factor Analysis**

| **Notes** | | |
| --- | --- | --- |
| Output Created | | 05-FEB-2022 08:55:01 |
| Comments | |  |
| Input | Data | C:\Users\Admin\Documents\Sagal thesis SPSS data Analysis UPDATED\sagal MSc thesis DATA.sav |
|  | Active Dataset | DataSet1 |
|  | File Label | EPIDATA FI THESIS by SAGAL'S MSc 630 DATA |
|  | Filter | <none> |
|  | Weight | <none> |
|  | Split File | <none> |
|  | N of Rows in Working Data File | 630 |
| Missing Value Handling | Definition of Missing | MISSING=EXCLUDE: User-defined missing values are treated as missing. |
|  | Cases Used | LISTWISE: Statistics are based on cases with no missing values for any variable used. |
| Syntax | | FACTOR  /VARIABLES W2WorkiRadio W3Refrigertor W4Car W6BankAccunt  /MISSING LISTWISE  /ANALYSIS W2WorkiRadio W3Refrigertor W4Car W6BankAccunt  /PRINT UNIVARIATE INITIAL CORRELATION KMO AIC EXTRACTION ROTATION  /FORMAT SORT BLANK(.30)  /PLOT EIGEN  /CRITERIA MINEIGEN(1) ITERATE(25)  /EXTRACTION PC  /CRITERIA ITERATE(25)  /ROTATION VARIMAX  /METHOD=CORRELATION. |
| Resources | Processor Time | 00:00:00.22 |
|  | Elapsed Time | 00:00:00.20 |
|  | Maximum Memory Required | 3008 (2.938K) bytes |

| **Descriptive Statistics** | | | |
| --- | --- | --- | --- |
|  | Mean | Std. Deviation | Analysis N |
| Radio | .26 | .440 | 630 |
| Refrigerator | .50 | .500 | 630 |
| Car | .21 | .407 | 630 |
| Bank account | .40 | .490 | 630 |

| **Correlation Matrix** | | | | | |
| --- | --- | --- | --- | --- | --- |
|  | | Radio | Refrigerator | Car | Bank account |
| Correlation | Radio | 1.000 | .596 | .598 | .196 |
|  | Refrigerator | .596 | 1.000 | .515 | .130 |
|  | Car | .598 | .515 | 1.000 | .109 |
|  | Bank account | .196 | .130 | .109 | 1.000 |

| **KMO and Bartlett's Test** | | |
| --- | --- | --- |
| Kaiser-Meyer-Olkin Measure of Sampling Adequacy. | | .709 |
| Bartlett's Test of Sphericity | Approx. Chi-Square | 616.333 |
|  | df | 6 |
|  | Sig. | .000 |

| **Anti-image Matrices** | | | | | |
| --- | --- | --- | --- | --- | --- |
|  | | Radio | Refrigerator | Car | Bank account |
| Anti-image Covariance | Radio | .519 | -.231 | -.236 | -.100 |
|  | Refrigerator | -.231 | .606 | -.149 | -.015 |
|  | Car | -.236 | -.149 | .603 | .012 |
|  | Bank account | -.100 | -.015 | .012 | .961 |
| Anti-image Correlation | Radio | .672^a^ | -.412 | -.421 | -.141 |
|  | Refrigerator | -.412 | .734^a^ | -.247 | -.020 |
|  | Car | -.421 | -.247 | .727^a^ | .015 |
|  | Bank account | -.141 | -.020 | .015 | .765^a^ |
| a. Measures of Sampling Adequacy(MSA) | | | | | |

| **Communalities** | | |
| --- | --- | --- |
|  | Initial | Extraction |
| Radio | 1.000 | .757 |
| Refrigerator | 1.000 | .673 |
| Car | 1.000 | .669 |
| Bank account | 1.000 | .094 |
| Extraction Method: Principal Component Analysis. | | |

| **Total Variance Explained** | | | | | | |
| --- | --- | --- | --- | --- | --- | --- |
| Component | Initial Eigenvalues | | | Extraction Sums of Squared Loadings | | |
|  | Total | % of Variance | Cumulative % | Total | % of Variance | Cumulative % |
| 1 | 2.193 | 54.831 | 54.831 | 2.193 | 54.831 | 54.831 |
| 2 | .953 | 23.816 | 78.647 |  |  |  |
| 3 | .485 | 12.119 | 90.766 |  |  |  |
| 4 | .369 | 9.234 | 100.000 |  |  |  |
| Extraction Method: Principal Component Analysis. | | | | | | |


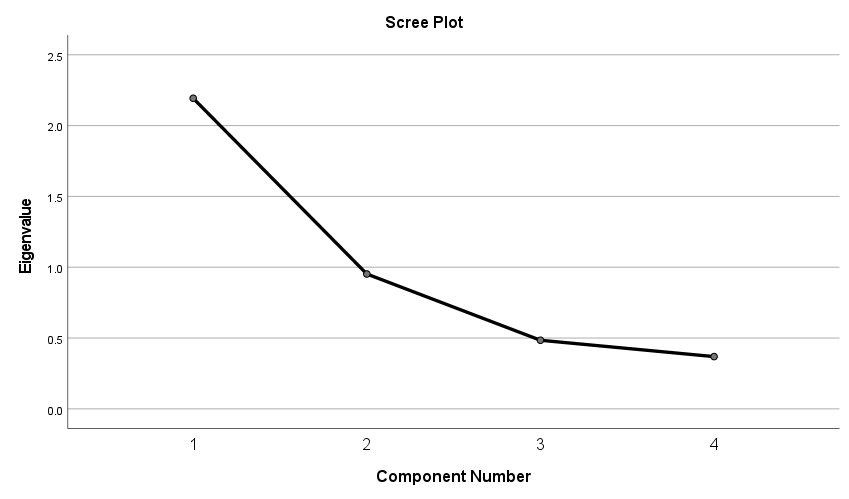


| **Component Matrix^a^** | |
| --- | --- |
|  | Component |
|  | 1 |
| Radio | .870 |
| Refrigerator | .821 |
| Car | .818 |
| Bank account | .306 |
| Extraction Method: Principal Component Analysis. | |
| a. 1 components extracted. | |

| **Rotated Component Matrix^a^** |
| --- |
|  |
| a. Only one component was extracted. The solution cannot be rotated. |

FACTOR

/VARIABLES W2WorkiRadio W3Refrigertor W4Car

/MISSING LISTWISE

/ANALYSIS W2WorkiRadio W3Refrigertor W4Car

/PRINT UNIVARIATE INITIAL CORRELATION KMO AIC EXTRACTION ROTATION

/FORMAT SORT BLANK(.30)

/CRITERIA MINEIGEN(1) ITERATE(25)

/EXTRACTION PC

/CRITERIA ITERATE(25)

/ROTATION VARIMAX

/METHOD=CORRELATION.

**Factor Analysis**

| **Notes** | | |
| --- | --- | --- |
| Output Created | | 05-FEB-2022 08:55:24 |
| Comments | |  |
| Input | Data | C:\Users\Admin\Documents\Sagal thesis SPSS data Analysis UPDATED\sagal MSc thesis DATA.sav |
|  | Active Dataset | DataSet1 |
|  | File Label | EPIDATA FI THESIS by SAGAL'S MSc 630 DATA |
|  | Filter | <none> |
|  | Weight | <none> |
|  | Split File | <none> |
|  | N of Rows in Working Data File | 630 |
| Missing Value Handling | Definition of Missing | MISSING=EXCLUDE: User-defined missing values are treated as missing. |
|  | Cases Used | LISTWISE: Statistics are based on cases with no missing values for any variable used. |
| Syntax | | FACTOR  /VARIABLES W2WorkiRadio W3Refrigertor W4Car  /MISSING LISTWISE  /ANALYSIS W2WorkiRadio W3Refrigertor W4Car  /PRINT UNIVARIATE INITIAL CORRELATION KMO AIC EXTRACTION ROTATION  /FORMAT SORT BLANK(.30)  /PLOT EIGEN  /CRITERIA MINEIGEN(1) ITERATE(25)  /EXTRACTION PC  /CRITERIA ITERATE(25)  /ROTATION VARIMAX  /METHOD=CORRELATION. |
| Resources | Processor Time | 00:00:00.47 |
|  | Elapsed Time | 00:00:00.37 |
|  | Maximum Memory Required | 1984 (1.938K) bytes |

| **Descriptive Statistics** | | | |
| --- | --- | --- | --- |
|  | Mean | Std. Deviation | Analysis N |
| Radio | .26 | .440 | 630 |
| Refrigerator | .50 | .500 | 630 |
| Car | .21 | .407 | 630 |

| **Correlation Matrix** | | | | |
| --- | --- | --- | --- | --- |
|  | | Radio | Refrigerator | Car |
| Correlation | Radio | 1.000 | .596 | .598 |
|  | Refrigerator | .596 | 1.000 | .515 |
|  | Car | .598 | .515 | 1.000 |

| **KMO and Bartlett's Test** | | |
| --- | --- | --- |
| Kaiser-Meyer-Olkin Measure of Sampling Adequacy. | | .702 |
| Bartlett's Test of Sphericity | Approx. Chi-Square | 591.837 |
|  | df | 3 |
|  | Sig. | .000 |

| **Anti-image Matrices** | | | | |
| --- | --- | --- | --- | --- |
|  | | Radio | Refrigerator | Car |
| Anti-image Covariance | Radio | .530 | -.237 | -.239 |
|  | Refrigerator | -.237 | .606 | -.149 |
|  | Car | -.239 | -.149 | .603 |
| Anti-image Correlation | Radio | .668^a^ | -.419 | -.423 |
|  | Refrigerator | -.419 | .724^a^ | -.246 |
|  | Car | -.423 | -.246 | .722^a^ |
| a. Measures of Sampling Adequacy(MSA) | | | | |

| **Communalities** | | |
| --- | --- | --- |
|  | Initial | Extraction |
| Radio | 1.000 | .758 |
| Refrigerator | 1.000 | .690 |
| Car | 1.000 | .692 |
| Extraction Method: Principal Component Analysis. | | |

| **Total Variance Explained** | | | | | | |
| --- | --- | --- | --- | --- | --- | --- |
| Component | Initial Eigenvalues | | | Extraction Sums of Squared Loadings | | |
|  | Total | % of Variance | Cumulative % | Total | % of Variance | Cumulative % |
| 1 | 2.140 | 71.332 | 71.332 | 2.140 | 71.332 | 71.332 |
| 2 | .485 | 16.173 | 87.505 |  |  |  |
| 3 | .375 | 12.495 | 100.000 |  |  |  |
| Extraction Method: Principal Component Analysis. | | | | | | |

| **Component Matrix^a^** | |
| --- | --- |
|  | Component |
|  | 1 |
| Radio | .871 |
| Car | .832 |
| Refrigerator | .831 |
| Extraction Method: Principal Component Analysis. | |
| a. 1 components extracted. | |

| **Rotated Component Matrix^a^** |
| --- |
|  |
| a. Only one component was extracted. The solution cannot be rotated. |

FACTOR

/VARIABLES W2WorkiRadio W3Refrigertor W4Car

/MISSING LISTWISE

/ANALYSIS W2WorkiRadio W3Refrigertor W4Car

/PRINT UNIVARIATE INITIAL CORRELATION KMO AIC EXTRACTION ROTATION

/FORMAT SORT BLANK(.30)

/CRITERIA MINEIGEN(1) ITERATE(25)

/EXTRACTION PC

/CRITERIA ITERATE(25)

/ROTATION VARIMAX

/SAVE REG(ALL)

/METHOD=CORRELATION.

**Factor Analysis**

| **Notes** | | |
| --- | --- | --- |
| Output Created | | 05-FEB-2022 08:56:13 |
| Comments | |  |
| Input | Data | C:\Users\Admin\Documents\Sagal thesis SPSS data Analysis UPDATED\sagal MSc thesis DATA.sav |
|  | Active Dataset | DataSet1 |
|  | File Label | EPIDATA FI THESIS by SAGAL'S MSc 630 DATA |
|  | Filter | <none> |
|  | Weight | <none> |
|  | Split File | <none> |
|  | N of Rows in Working Data File | 630 |
| Missing Value Handling | Definition of Missing | MISSING=EXCLUDE: User-defined missing values are treated as missing. |
|  | Cases Used | LISTWISE: Statistics are based on cases with no missing values for any variable used. |
| Syntax | | FACTOR  /VARIABLES W2WorkiRadio W3Refrigertor W4Car  /MISSING LISTWISE  /ANALYSIS W2WorkiRadio W3Refrigertor W4Car  /PRINT UNIVARIATE INITIAL CORRELATION KMO AIC EXTRACTION ROTATION  /FORMAT SORT BLANK(.30)  /PLOT EIGEN  /CRITERIA MINEIGEN(1) ITERATE(25)  /EXTRACTION PC  /CRITERIA ITERATE(25)  /ROTATION VARIMAX  /SAVE REG(ALL)  /METHOD=CORRELATION. |
| Resources | Processor Time | 00:00:00.45 |
|  | Elapsed Time | 00:00:00.29 |
|  | Maximum Memory Required | 2184 (2.133K) bytes |
| Variables Created | FAC1_1 | Component score 1 |

| **Descriptive Statistics** | | | |
| --- | --- | --- | --- |
|  | Mean | Std. Deviation | Analysis N |
| Radio | .26 | .440 | 630 |
| Refrigerator | .50 | .500 | 630 |
| Car | .21 | .407 | 630 |

| **Correlation Matrix** | | | | |
| --- | --- | --- | --- | --- |
|  | | Radio | Refrigerator | Car |
| Correlation | Radio | 1.000 | .596 | .598 |
|  | Refrigerator | .596 | 1.000 | .515 |
|  | Car | .598 | .515 | 1.000 |

| **KMO and Bartlett's Test** | | |
| --- | --- | --- |
| Kaiser-Meyer-Olkin Measure of Sampling Adequacy. | | .702 |
| Bartlett's Test of Sphericity | Approx. Chi-Square | 591.837 |
|  | df | 3 |
|  | Sig. | .000 |

| **Anti-image Matrices** | | | | |
| --- | --- | --- | --- | --- |
|  | | Radio | Refrigerator | Car |
| Anti-image Covariance | Radio | .530 | -.237 | -.239 |
|  | Refrigerator | -.237 | .606 | -.149 |
|  | Car | -.239 | -.149 | .603 |
| Anti-image Correlation | Radio | .668^a^ | -.419 | -.423 |
|  | Refrigerator | -.419 | .724^a^ | -.246 |
|  | Car | -.423 | -.246 | .722^a^ |
| a. Measures of Sampling Adequacy(MSA) | | | | |

| **Communalities** | | |
| --- | --- | --- |
|  | Initial | Extraction |
| Radio | 1.000 | .758 |
| Refrigerator | 1.000 | .690 |
| Car | 1.000 | .692 |
| Extraction Method: Principal Component Analysis. | | |

| **Total Variance Explained** | | | | | | |
| --- | --- | --- | --- | --- | --- | --- |
| Component | Initial Eigenvalues | | | Extraction Sums of Squared Loadings | | |
|  | Total | % of Variance | Cumulative % | Total | % of Variance | Cumulative % |
| 1 | 2.140 | 71.332 | 71.332 | 2.140 | 71.332 | 71.332 |
| 2 | .485 | 16.173 | 87.505 |  |  |  |
| 3 | .375 | 12.495 | 100.000 |  |  |  |
| Extraction Method: Principal Component Analysis. | | | | | | |

| **Component Matrix^a^** | |
| --- | --- |
|  | Component |
|  | 1 |
| Radio | .871 |
| Car | .832 |
| Refrigerator | .831 |
| Extraction Method: Principal Component Analysis. | |
| a. 1 components extracted. | |

| **Rotated Component Matrix^a^** |
| --- |
|  |
| a. Only one component was extracted. The solution cannot be rotated. |

RANK VARIABLES=FAC1_1 (A)

/NTILES(3)

/PRINT=YES

/TIES=MEAN.

**RANK**

| **Notes** | | |
| --- | --- | --- |
| Output Created | | 05-FEB-2022 08:57:11 |
| Comments | |  |
| Input | Data | C:\Users\Admin\Documents\Sagal thesis SPSS data Analysis UPDATED\sagal MSc thesis DATA.sav |
|  | Active Dataset | DataSet1 |
|  | File Label | EPIDATA FI THESIS by SAGAL'S MSc 630 DATA |
|  | Filter | <none> |
|  | Weight | <none> |
|  | Split File | <none> |
|  | N of Rows in Working Data File | 630 |
| Missing Value Handling | Definition of Missing | User-defined missing values are treated as missing. |
|  | Cases Used | All non-missing data are used. |
| Syntax | | RANK VARIABLES=FAC1_1 (A)  /NTILES(3)  /PRINT=YES  /TIES=MEAN. |
| Resources | Processor Time | 00:00:00.02 |
|  | Elapsed Time | 00:00:00.02 |
| Variables Created or Modified | NFAC1_1 | Percentile Group of FAC1_1 |

| **Created Variables^a^** | | | |
| --- | --- | --- | --- |
| Source Variable | Function | New Variable | Label |
| FAC1_1^c^ | Percentile Group^b^ | NFAC1_1 | Percentile Group of FAC1_1 |
| a. Mean rank of tied values is used for ties. | | | |
| b. 3 groups are generated. | | | |
| c. Ranks are in ascending order. | | | |

FREQUENCIES VARIABLES=NFAC1_1

/STATISTICS=RANGE MINIMUM MAXIMUM MEDIAN MEAN MODE

/ORDER=ANALYSIS.

**Frequencies**

| **Notes** | | |
| --- | --- | --- |
| Output Created | | 05-FEB-2022 08:57:18 |
| Comments | |  |
| Input | Data | C:\Users\Admin\Documents\Sagal thesis SPSS data Analysis UPDATED\sagal MSc thesis DATA.sav |
|  | Active Dataset | DataSet1 |
|  | File Label | EPIDATA FI THESIS by SAGAL'S MSc 630 DATA |
|  | Filter | <none> |
|  | Weight | <none> |
|  | Split File | <none> |
|  | N of Rows in Working Data File | 630 |
| Missing Value Handling | Definition of Missing | User-defined missing values are treated as missing. |
|  | Cases Used | Statistics are based on all cases with valid data. |
| Syntax | | FREQUENCIES VARIABLES=NFAC1_1  /STATISTICS=RANGE MINIMUM MAXIMUM MEDIAN MEAN MODE  /ORDER=ANALYSIS. |
| Resources | Processor Time | 00:00:00.00 |
|  | Elapsed Time | 00:00:00.00 |

| **Statistics** | | |
| --- | --- | --- |
| Percentile Group of FAC1_1 | | |
| N | Valid | 630 |
|  | Missing | 0 |
| Mean | | 1.81 |
| Median | | 1.50 |
| Mode | | 1 |
| Range | | 2 |
| Minimum | | 1 |
| Maximum | | 3 |

| **Percentile Group of FAC1_1** | | | | | |
| --- | --- | --- | --- | --- | --- |
|  | | Frequency | Percent | Valid Percent | Cumulative Percent |
| Valid | 1 | 315 | 50.0 | 50.0 | 50.0 |
|  | 2 | 120 | 19.0 | 19.0 | 69.0 |
|  | 3 | 195 | 31.0 | 31.0 | 100.0 |
|  | Total | 630 | 100.0 | 100.0 |  |

DATASET ACTIVATE DataSet1.

SAVE OUTFILE='C:\Users\Admin\Documents\Sagal thesis SPSS data Analysis UPDATED\sagal MSc thesis '+

'DATA.sav'

/COMPRESSED.

RANK VARIABLES=Wealth_Index (A)

/NTILES(3)

/PRINT=YES

/TIES=MEAN.

**RANK**

| **Notes** | | |
| --- | --- | --- |
| Output Created | | 05-FEB-2022 09:02:08 |
| Comments | |  |
| Input | Data | C:\Users\Admin\Documents\Sagal thesis SPSS data Analysis UPDATED\sagal MSc thesis DATA.sav |
|  | Active Dataset | DataSet1 |
|  | File Label | EPIDATA FI THESIS by SAGAL'S MSc 630 DATA |
|  | Filter | <none> |
|  | Weight | <none> |
|  | Split File | <none> |
|  | N of Rows in Working Data File | 630 |
| Missing Value Handling | Definition of Missing | User-defined missing values are treated as missing. |
|  | Cases Used | All non-missing data are used. |
| Syntax | | RANK VARIABLES=Wealth_Index (A)  /NTILES(3)  /PRINT=YES  /TIES=MEAN. |
| Resources | Processor Time | 00:00:00.02 |
|  | Elapsed Time | 00:00:00.02 |
| Variables Created or Modified | NWealth_ | Percentile Group of Wealth_Index |

| **Created Variables^a^** | | | |
| --- | --- | --- | --- |
| Source Variable | Function | New Variable | Label |
| Wealth_Index^c^ | Percentile Group^b^ | NWealth_ | Percentile Group of Wealth_Index |
| a. Mean rank of tied values is used for ties. | | | |
| b. 3 groups are generated. | | | |
| c. Ranks are in ascending order. | | | |

RANK VARIABLES=Incomescale (A)

/NTILES(3)

/PRINT=YES

/TIES=MEAN.

**RANK**

| **Notes** | | |
| --- | --- | --- |
| Output Created | | 05-FEB-2022 09:03:03 |
| Comments | |  |
| Input | Data | C:\Users\Admin\Documents\Sagal thesis SPSS data Analysis UPDATED\sagal MSc thesis DATA.sav |
|  | Active Dataset | DataSet1 |
|  | File Label | EPIDATA FI THESIS by SAGAL'S MSc 630 DATA |
|  | Filter | <none> |
|  | Weight | <none> |
|  | Split File | <none> |
|  | N of Rows in Working Data File | 630 |
| Missing Value Handling | Definition of Missing | User-defined missing values are treated as missing. |
|  | Cases Used | All non-missing data are used. |
| Syntax | | RANK VARIABLES=Incomescale (A)  /NTILES(3)  /PRINT=YES  /TIES=MEAN. |
| Resources | Processor Time | 00:00:00.00 |
|  | Elapsed Time | 00:00:00.05 |
| Variables Created or Modified | NIncomes | Percentile Group of Incomescale |

| **Created Variables^a^** | | | |
| --- | --- | --- | --- |
| Source Variable | Function | New Variable | Label |
| Incomescale^c^ | Percentile Group^b^ | NIncomes | Percentile Group of Incomescale |
| a. Mean rank of tied values is used for ties. | | | |
| b. 3 groups are generated. | | | |
| c. Ranks are in ascending order. | | | |

SORT CASES BY NIncomes (A).

FREQUENCIES VARIABLES=NIncomes

/STATISTICS=RANGE MINIMUM MAXIMUM MEDIAN MEAN MODE

/ORDER=ANALYSIS.

**Frequencies**

| **Notes** | | |
| --- | --- | --- |
| Output Created | | 05-FEB-2022 09:03:19 |
| Comments | |  |
| Input | Data | C:\Users\Admin\Documents\Sagal thesis SPSS data Analysis UPDATED\sagal MSc thesis DATA.sav |
|  | Active Dataset | DataSet1 |
|  | File Label | EPIDATA FI THESIS by SAGAL'S MSc 630 DATA |
|  | Filter | <none> |
|  | Weight | <none> |
|  | Split File | <none> |
|  | N of Rows in Working Data File | 630 |
| Missing Value Handling | Definition of Missing | User-defined missing values are treated as missing. |
|  | Cases Used | Statistics are based on all cases with valid data. |
| Syntax | | FREQUENCIES VARIABLES=NIncomes  /STATISTICS=RANGE MINIMUM MAXIMUM MEDIAN MEAN MODE  /ORDER=ANALYSIS. |
| Resources | Processor Time | 00:00:00.02 |
|  | Elapsed Time | 00:00:00.02 |

| **Statistics** | | |
| --- | --- | --- |
| Percentile Group of Incomescale | | |
| N | Valid | 630 |
|  | Missing | 0 |
| Mean | | 1.99 |
| Median | | 2.00 |
| Mode | | 1 |
| Range | | 2 |
| Minimum | | 1 |
| Maximum | | 3 |

| **Percentile Group of Incomescale** | | | | | |
| --- | --- | --- | --- | --- | --- |
|  | | Frequency | Percent | Valid Percent | Cumulative Percent |
| Valid | 1 | 220 | 34.9 | 34.9 | 34.9 |
|  | 2 | 194 | 30.8 | 30.8 | 65.7 |
|  | 3 | 216 | 34.3 | 34.3 | 100.0 |
|  | Total | 630 | 100.0 | 100.0 |  |

FREQUENCIES VARIABLES=NWealth_

/STATISTICS=RANGE MINIMUM MAXIMUM MEDIAN MEAN MODE

/ORDER=ANALYSIS.

**Frequencies**

| **Notes** | | |
| --- | --- | --- |
| Output Created | | 05-FEB-2022 10:16:20 |
| Comments | |  |
| Input | Data | C:\Users\Admin\Documents\Sagal thesis SPSS data Analysis UPDATED\sagal MSc thesis DATA.sav |
|  | Active Dataset | DataSet1 |
|  | File Label | EPIDATA FI THESIS by SAGAL'S MSc 630 DATA |
|  | Filter | <none> |
|  | Weight | <none> |
|  | Split File | <none> |
|  | N of Rows in Working Data File | 630 |
| Missing Value Handling | Definition of Missing | User-defined missing values are treated as missing. |
|  | Cases Used | Statistics are based on all cases with valid data. |
| Syntax | | FREQUENCIES VARIABLES=NWealth_  /STATISTICS=RANGE MINIMUM MAXIMUM MEDIAN MEAN MODE  /ORDER=ANALYSIS. |
| Resources | Processor Time | 00:00:00.00 |
|  | Elapsed Time | 00:00:00.00 |

| **Statistics** | | |
| --- | --- | --- |
| Percentile Group of Wealth_Index | | |
| N | Valid | 630 |
|  | Missing | 0 |
| Mean | | 1.81 |
| Median | | 1.50 |
| Mode | | 1 |
| Range | | 2 |
| Minimum | | 1 |
| Maximum | | 3 |

| **Percentile Group of Wealth_Index** | | | | | |
| --- | --- | --- | --- | --- | --- |
|  | | Frequency | Percent | Valid Percent | Cumulative Percent |
| Valid | lower | 315 | 50.0 | 50.0 | 50.0 |
|  | middle | 120 | 19.0 | 19.0 | 69.0 |
|  | higher | 195 | 31.0 | 31.0 | 100.0 |
|  | Total | 630 | 100.0 | 100.0 |  |
